# Synthesis, characterization, and application of novel aryldiazenyl disperse dyes on polyester fabrics

**DOI:** 10.1038/s41598-023-48368-y

**Published:** 2023-12-06

**Authors:** Alaa Z. Omar, Asmaa M. Khamis, Ezzat A. Hamed, Samir K. El-Sadany, Elsayed M. Abdel Rehim, Mohamed E. Elba, Mohamed G. Mohamed, Mohamed A. El-Atawy

**Affiliations:** 1 Chemistry Department, Faculty of Science, Alexandria University, P.O. 426, Ibrahemia, Alexandria, 21321 Egypt; 2 Chemistry Department, Faculty of Science, Damanhour University, Damanhour, Egypt; 3 Chemistry Department, Faculty of Science, Taibah University, 46423 Yanbu, Saudi Arabia

**Keywords:** Organic chemistry, Chemical synthesis

## Abstract

Azo dyes are widely used for dyeing polyester fabrics but require optimization of properties like color strength and fastness. Fourteen novel disperse azo dyes were synthesized from 2,3-naphthalenediol and aniline derivatives to examine their potential for polyester dyeing. The dyes were prepared via diazotization and coupling reactions and characterized using FT-IR, UV–Vis, ^1^H NMR, ^13^C NMR, and elemental analysis. Furthermore, several techniques were employed to study the azo-hydrazone tautomerism, including UV–Vis spectroscopy, NMR spectroscopy, and computational methods. DFT computations revealed hydrazone tautomers were more stable than azo tautomers. The prepared azo dyes were applied on polyester fabrics at 2% depth using a high temperature pressure technique in water utilizing DYEWELL-002 as a dispersing agent. The color shading of dyed polyester samples ranged from peach amber to apple of my eye, depending on the coupler moieties. The fastness properties, assessed using a grey scale of dyed polyester fabrics, indicated very good to excellent grades for most dyes. Additionally, measurements of color strength (K/S), dye exhaustion (%E), as well as colorimetric colors CILAB of dyed polyester fabrics values, were measured and discussed in terms of the effect of substituents. The findings provide new insights into structure-performance relationships to design optimized disperse dyes for polyester coloration. Overall, the synthesized aryldiazenyl dyes are promising candidates for dyeing polyester fabrics across a spectrum of shades with good fastness properties.

## Introduction

2,3-Dihydroxynaphthalene (2,3-DHN) is a synthetic intermediate that is used in various applications, including the separation of certain metals, the production of dyes, pigments, and cosmetics, and the synthesis of antifungal and antibacterial compounds. The ability of complex formation of 2,3-DHN was used to extract V, W, Fe, Ti, and Mo complexes^1^. The formation of a 1:3 anionic complex between UO_2_^2+^ with 2,3-DHN was found capable of quantitatively separating even ultra-trace amounts of uranium from rock samples^2^. It has been reported that 2,3-DHN is complexed with Fe and is employed in various applications, including silicate rocks, soils, tobacco leaves, stream sediments, concentrates, cigarettes, minerals, and water^3^. Furthermore, 2,3-DHN is utilized in cosmetics as a component of oxidative hair dyes which have the potential to come in contact with skin and eyes^4^. Moreover, 2,3-DHN serves as a synthetic intermediate in the production of numerous dyes^5^. 2,3-DHN is also applied in oxidative coupling polymerization reactions^6^ and used as a synthetic intermediate to prepare crown ether^4^. Interestingly, derivatives of 2,3-DHN showed moderate antifungal activity against *Candida albicans* of considerable interest^7^.

Azo dyes are the most widely used dyes for textile and fiber dyeing, owing to their adsorption ability and exhaustion efficiency resulting from the presence of the –N=N– group^8–10^. The essential structural characteristics that determine the adsorption of these molecules onto the fiber surface are the planarity (π) and the presence of electrons lone pair on the *N* atoms of the azo group.

Azo-hydrazone tautomerism is a type of tautomerism in which azo compounds and hydrazones undergo interconversion. It involves the movement of electrons and a hydrogen atom within a molecule, leading to the formation of different isomers. Moreover, it plays a role in the synthesis of complex compounds and influences the properties of the resulting molecules.

The two isomeric forms of azo-hydrazone tautomers have different physicochemical properties and reactivity. The azo form is highly stable, possesses a relatively long conjugated system, and would show intense absorption in the visible region. In contrast, the hydrazone form is less stable than the azo form and has a shorter conjugated system and would exhibits absorption with a hypsochromic shift compared to the azo form.

The tautomeric equilibrium between the azo and hydrazone forms can be influenced by various factors, including the nature of the substituents, solvent polarity, pH, and temperature. Electron-donating substituents tend to shift the equilibrium towards the azo form, whereas an electron-withdrawing groups favor the hydrazone form^11^.

Polyethylene terephthalate (PET) is the most widely used fiber in the textile industry^12,13^. However, its compact, crystalline, and hydrophobic structure presents challenges in the dyeing process^14^. The dyeing of PET fabrics with disperse azo dyes proceeded either in a high-pressure vessel at a high temperature (130 °C)^15^ or in the presence of carriers for good dye ability at atmospheric pressure^16^. Moreover, the dyeing process of PET has garnered significant attention due to its environmental impact. Researchers have been exploring eco-friendly dyeing techniques to mitigate the ecological footprint associated with traditional high-temperature processes. In microencapsulation, the dye is encapsulated in tiny polymer capsules^17^. These capsules are then applied to the fabric, and the dye is released gradually when the fabric is exposed to heat. This method is useful for achieving color change or pattern effects. Digital printing is a modern and highly efficient method for dyeing PET. In this process, the desired design is printed directly onto the fabric using inkjet technology. It allows for intricate designs and precise color placement, reducing water usage and waste compared to traditional dyeing methods^18^. Sublimation printing is a heat transfer method where the dye is sublimated into a gaseous state and then absorbed into the polyester fibers^19^. It's often used for sportswear and promotional items and provides vibrant, durable prints. Supercritical fluid dyeing process is an environmentally friendly method that uses supercritical carbon dioxide as a solvent to dissolve and transport the dye into the polyester fibers^20^. It offers reduced water and energy consumption and is considered a sustainable option. Ultrasonic dyeing uses high-frequency sound waves to facilitate the penetration of dye molecules into polyester fibers. It can reduce the dyeing time and improve color fastness. Each of these techniques has its advantages and limitations, and the choice of method depends on factors such as the desired color, fabric type, environmental considerations, and production requirements. Manufacturers may choose the most suitable technique based on these factors to achieve the desired results while minimizing environmental impact.

Most disperse dyes belong to the category of azo dyes, which can generate a wide range of molecular combinations by varying the diazo and coupling components and provide a high color gamut^21–23^. Disperse azo dyes are particularly popular for dyeing polyester fabrics due to their brilliance^24,25^, extensive color range, and outstanding fastness properties^26^.

In recent years, there has been significant progress in developing new disperse dye structures with improved properties for polyester dyeing. Several research groups have synthesized disperse dyes with modified molecular structures aimed at enhancing features like color fastness, intensity, and strength on polyesters. New disperse dyes, include pyrazole-based dyes^10^, dyes with long conjugation of naphthalene rings^27^, and dyes with additional azomethine group^23^, have shown excellent compatibility and affinity for dyeing polyester fabrics. These structural modifications have produced disperse dyes with excellent thermal stability, migration resistance, brilliant hues, and high tinctorial strength according to application studies.

The exhaustion effect is influenced by various physicochemical and electronic properties of organic dyes, including functional groups, electronic density on donor atoms, steric effects, and orbital character of donating electrons. The application of quantum chemical methods has proven useful in determining the molecular and electronic structures, as well as reactivity, of these dyes. As a result, it has become common to employ quantum chemical calculations in exhaustion studies^8–10^.

The purpose of this study is to further explore the synthesis and elucidation of the tautomeric structures of mono-azo dyes suitable for dyeing polyester fabrics^8–10^. Specifically, we will synthesize a series of azo disperse dyes, namely 1-(aryldiazenyl) naphthalene-2,3-diols with various substituted groups in the aryl moiety **1–14**, Scheme 1, in an effort to provide a better understanding of the prevailing tautomeric structure of these compounds.

**Figure 1:**
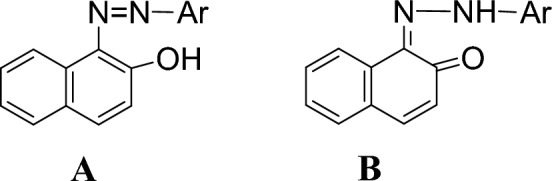
The tautomeric forms of aryl azo dyes with ortho hydroxyl group (**A**) Azo-form (**B**) Keto-hydrazone form.

The azo-hydrazone tautomeric behavior of these dyes has been extensively studied; due to its significance in various fields such as organic synthesis^28^, photochemistry, polymer chemistry, and pharmaceuticals. Figure 1 shows the two possible tautomeric forms namely, azo form (**A**)^29^ and keto-hydrazone form (**B**)^30^.

Additionally, we will evaluate the color strength properties (K/S), color intensity properties, the effect of auxochrome, fastness, and the position of color in CIELAB coordinates (L*, a*, b*, h*, c*) by dyeing the prepared dyestuffs onto polyester fabrics.

## Results and discussion

### Chemistry

#### Synthesis and characterizations

**Figure 2:**
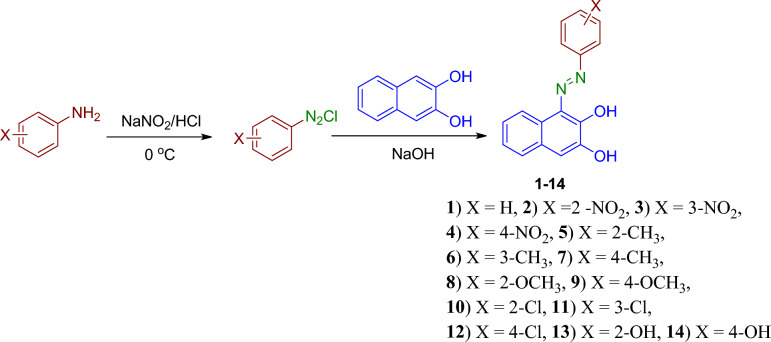
The Synthetic pathway of azo derivatives of 1-(aryldiazenyl)naphthalene-2,3-diols **1–14.**

1-(Aryldiazenyl)naphthalene-2,3-diols **1–14** were synthesized by the diazotization of aniline derivatives followed by coupling with 2,3-naphthalenediol, Fig. 2^5,31–33^. The progress of the reactions was monitored using thin-layer chromatography (TLC) to ensure their completion. Subsequently, the synthesized compounds were characterized using various techniques, including UV/Vis, FT-IR, and NMR spectroscopy (^1^H and ^13^C), to confirm their identity and purity. Additionally, DFT studies were employed to calculate the theoretical λ_max_ and assess the stability of the possible azo and hydrazone tautomeric form expected to coexist for the dyes **1–14**.

**Figure 3:**
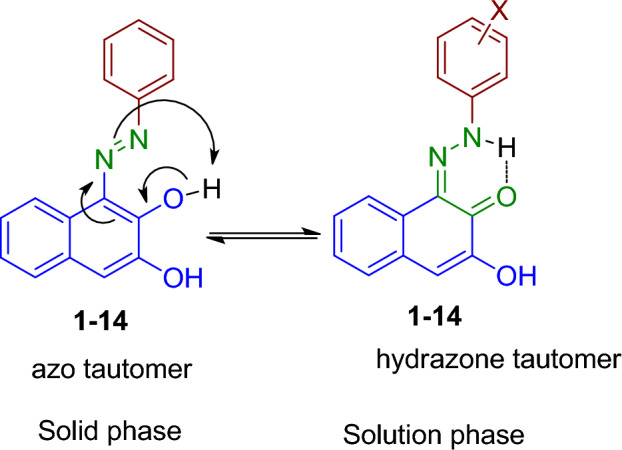
The azo-enol and keto-hydrazone tautomerism of synthesized dyes **1–14.**

The two isomeric forms of azo-hydrazone tautomers of disperse dyes 1–14 were presented in Fig. 3. In azo-hydrazone tautomerism, the azo group undergoes tautomerization due to the presence of a conjugated acidic proton (the hydroxyl proton at C_2_). The migration of this proton via the conjugated system results in the formation of a hydrazone group in which the N=N bond is replaced with a C=N bond. The hydrazone form has an intramolecular hydrogen bond between N–H of the hydrazone group and *ortho*-keto functional group with the formation of a stable six-membered ring^28,30^, Fig. 3**.**

Several techniques have been employed to investigate the azo-hydrazone tautomerism, including UV–Vis spectroscopy, NMR spectroscopy, X-ray crystallography, and computational methods. These studies have provided important insights into the mechanism and kinetics of the tautomeric interconversion, as well as their impact on the chemical and biological properties of the molecules.

The FT-IR spectra of dyes **1–14** showed a broad band in the region of 3203–3448 cm^−1^ corresponding to characteristic O–H stretching. Moreover, the spectra displayed aromatic (=C–H) stretching absorption bands at 3030–3171 cm^−1^, while dyes **5–9** exhibited an additional aliphatic (C–H) stretching bands at 2919–2974 cm^−1^due to presence of methyl group. Furthermore, the IR spectra revealed a characteristic absorption band at 1596–1517 cm^−1^ which has been assigned to azo group (N=N). The nitro group (NO_2_) of compounds **2–4** showed peaks at 1525–1509 and 1347–1317 cm^−1^ that has been assigned to the characteristic asymmetric and symmetric stretching of (N–O), respectively. The absence of the characteristic absorption band for carbonyl group (C=O) confirms the absence of the hydrazone tautmer in the solid phase.

In contrast the FT-IR for the dyed fiber with dye **1** (as a porotype) showed peaks at wave number 3646, 3340 and 1691 cm^−1^ corresponding to –OH, NH and C=O groups, respectively, and not peak related to azo –N=N–. This observation suggests the existence of the chromophore of hydrazone form persisted in the dyed polyester fabrics.

^1^H NMR of dyes **1–5** and **10–14** has been recorded in deuterated dimethyl sulfoxide while dyes **6–9** were recorded in deuterated chloroform. the spectrum exhibited two sets of signals for two different aromatic protons at the region δ 8.80–6.82 ppm that corresponds to naphthyl and aryl protons. Moreover, two singlet signals appeared at ≈ 16 ppm and 9 ppm for two exchangeable protons has been assigned for OH and NH, respectively. The highly deshielded OH signal within 16 ppm is consistent with hydrazone form not the azo form. Furthermore, ^13^C NMR exhibited peaks at 171.17–174.34 ppm that has been assigned to the carbonyl group^34^, which confirms the predominance of the hydrazone form in the solution.

#### Ultraviolet–visible characteristics of azo disperse dyes 1–14

The UV spectral characteristics of phenylazo-2,3-dihydroxy naphthalene **1** are analogous to those of (4-arylazo-2,3-dihydroxy naphthalene) dyes **2–14**. UV–Vis spectroscopy can be employed to study the azo-hydrazone tautomeric equilibrium. Due to the differences in the electronic structures of the two isomeric forms, they exhibit different UV–Vis absorption spectra. UV–Vis spectra were recorded for the dyes under investigation in DMF as a solvent, and the molar absorptivity has been reported in the experimental section. Two possible transitions are expected for the investigated dyes **1–14** (the π → π ∗ and n → π ∗), which, however, depend on the class of the chromophore, the nature of auxochrome, and the possibility of the existence of more than one tautomer. The presence of two adjacent OH groups in the same aromatic ring and a stable azo chromophoric group (–N=N–) conjugated with an aromatic ring results in a molecule with intense and highly stable color throughout the visible region of light^35–38^.

**Figure 4:**
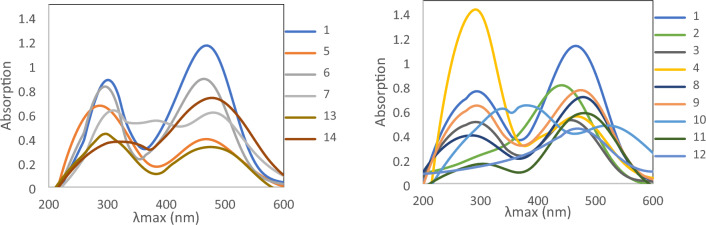
UV–Visible absorption spectra of dyes **1–14** in DMF.

As shown in Table 1, in a preliminary examination of dyes **1–14** revealed, as expected, two main bands, Fig. 4. The first band, observed at wavelength 270–290 nm, and was assigned to the low-energy π → π ∗ transition. The second band within the range 435–520 nm is analogous to that reported for the chromophoric hydrazone group's n-π* transition (K bands). Whereas some dyes' have UV absorptions arising from the azo group (N=N) at around 300–375 nm^39^, Table 1 probably suggests the coexistence of both azo and hydrazone tautomers in DMF.

**Table 1:** The calculated (hydrazone and azo) and experimental absorption maxima (**λ**_**max**_) of azo dyes **1–14** in DMF, DMF/0.1 M HCl and DMF/0.1 M NaOH.

Dye	X	λ_max_ (experimental)	λ_max_ (theoretical)		λ_max_ (HCl)	λ_max_ (NaOH)
			Hydrazone	Azo		
1	–H	275, 305, 470	290, 467	300, 410	465	440
2	2–NO_2_	285, 435	320, 501	240	400	400
3	3–NO_2_	280, 295, 465	320, 481	300, 425	465	460
4	4–NO_2_	290, 470	330, 518	320, 485	450	400
5	2–Cl	285, 470	280, 466	300, 409	470	400
6	3–Cl	275, 300, 465	280, 475	310, 420	465	460
7	4–Cl	280, 375, 480	270, 474	250, 320, 415	465	395
8	2–CH_3_	275, 305, 480	290, 461	300, 400	480	465
9	3–CH_3_	280, 305, 475	290, 466	250, 310, 410	475	445
10	4–CH_3_	375, 520	290, 466	250, 320, 410	430	440
11	2–OCH_3_	315, 480	290, 464	290, 410	455	450
12	4–OCH_3_	470	270, 475	250, 330, 410	465	440
13	2–OH	310, 475	280, 459	300, 415	405	455
14	4–OH	320, 480	270, 472	250, 320, 410	470	435

Furthermore, UV theoretical calculations using B3LYP/6-31G (d,p)-TD-DFT validated the experimental findings. Table 1 displays the DMF-UV calculated absorption spectra for the two expected tautomers of compounds **1–14**. The results demonstrate that the calculated band values for the azo and hydrazone forms are within the bands of practically relevant measurements, suggesting the presence of tautomerism between the azo and hydrazone tautomers in DMF.

The value of λ_max_ for dyes **1–14** relatively depends on the position and nature of substituent groups X group in the aryl system.

The electronic effects of substituents on the λ_max_ is a well-known phenomenon in azo dye molecules^40^. The introduction of an electron-acceptor substituent, such as nitro and chloro groups in various positions (*ortho* -, *meta*—and *para* -) of the phenyl ring, causes small hypsochromic shifts compared to unsubstituted dye **1** except for dye** 2** (X = 2–NO_2_) which exhibits a hypsochromic shift of about Δλ = 35 nm**.** Conversely, introducing an electron-donating group, such as methyl, methoxyl, or hydroxyl in the phenyl ring, results in a slight bathochromic shift compared to the unsubstituted analogous **1.** A strong bathochromic shift of about Δλ = 50 nm was reported for the derivative dye **10** (X = 4–CH_3_), which has a larger λ_max_ than most others.

**Figure 5:**
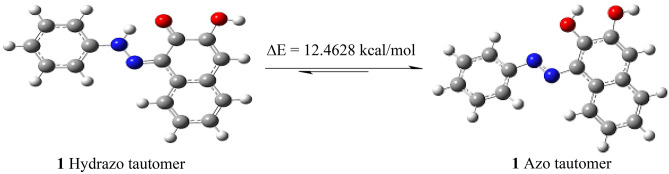
Geometrical optimization of the hydrazo-azo tautomers of dye **1.**

Finally, DFT Theoretical calculation has been employed to calculate the tautomerization energy for the two tautomers of dyes **1–14** in gas phase^41^, Fig. 5. The geometric optimization of dyes **1–14** revealed that the hydrazone tautomer is more stable, with lower relative energy (∆E = 11.1821–51.2319 kcal/mol) relative to the azo tautomer, Table 2. However, Azo form of dye **3** is more stable than the hydrazone tautomer by ∆E = 11.2951 kcal/mol.

**Table 2:** The energy of tautomers (azo and hydrazo) of dyes **1–14** were calculated by DFT using B3LYP/6-31G(d,p).

Dye number	Azo E (Hartree)	Hydrazo E (Hartree)	∆E (kcal/mol)
1	− 876.857674	− 876.877535	12.4628
2	− 1081.280220	− 1081.361864	51.2319
3	− 1081.357458	− 1081.375886	11.2951
4	− 1081.358200	− 1081.378290	12.6065
5	− 1336.442372	− 1336.460192	11.1821
6	− 1336.452100	− 1336.471162	11.9615
7	− 1336.452500	− 1336.471253	11.7676
8	− 916.173556	− 916.193093	12.2595
9	− 916.178183	− 916.198327	12.6404
10	− 916.178659	− 916.198229	12.2802
11	− 991.374503	− 991.392942	11.5705
12	− 991.384259	− 991.402329	11.339
13	− 952.090772	− 952.102747	7.51435
14	− 952.079270	− 952.097073	11.1714

#### Effect of acid and base

The effect of an acid or base on the UV spectra of dyes that undergo azo-hydrazone tautomersim depends on the specific structure of the dye and the strength of the acid or base. Moreover, interpreting UV spectra in the presence of acid or base can provide valuable information about the chemical behavior and stability of dyes. The results of adding an acid or base to the absorption spectra of the dye solutions are summarized in Table 1. The absorption spectra of dyes **1–14** in DMF responded to the addition of a base (sodium hydroxide, 0.1 M). Moreover, the λ_max_ of dyes **1–14** are bule shifted upon adding the base to DMF, compared to the corresponding dye in DMF only. For instance, λ_max_ of dye **1** was observed at 470 nm in DMF and 440 in DMF in the presence of NaOH, whereas λ_max_ of dye **10** was recorded at 525 nm in DMF and 400 nm in DMF + NaOH. In contrast, when hydrochloric acid (0.1 M) was added to the dye solutions in DMF, slight hyposchromic shifts or no change in λ_max_ was observed. The λ_max_ of **1–14** was observed at 435–520 nm in DMF and 400–475 nm in DMF + HCl.

The shift in the UV spectra towards longer wavelengths could be due to predominance of azo form because the conjugation is extended. Conversely, a shift in the UV spectra towards shorter wavelengths may attributed to predominance of hydrazone tautomer because the conjugation is disrupted.

### Visual assessment

**Figure 6:**
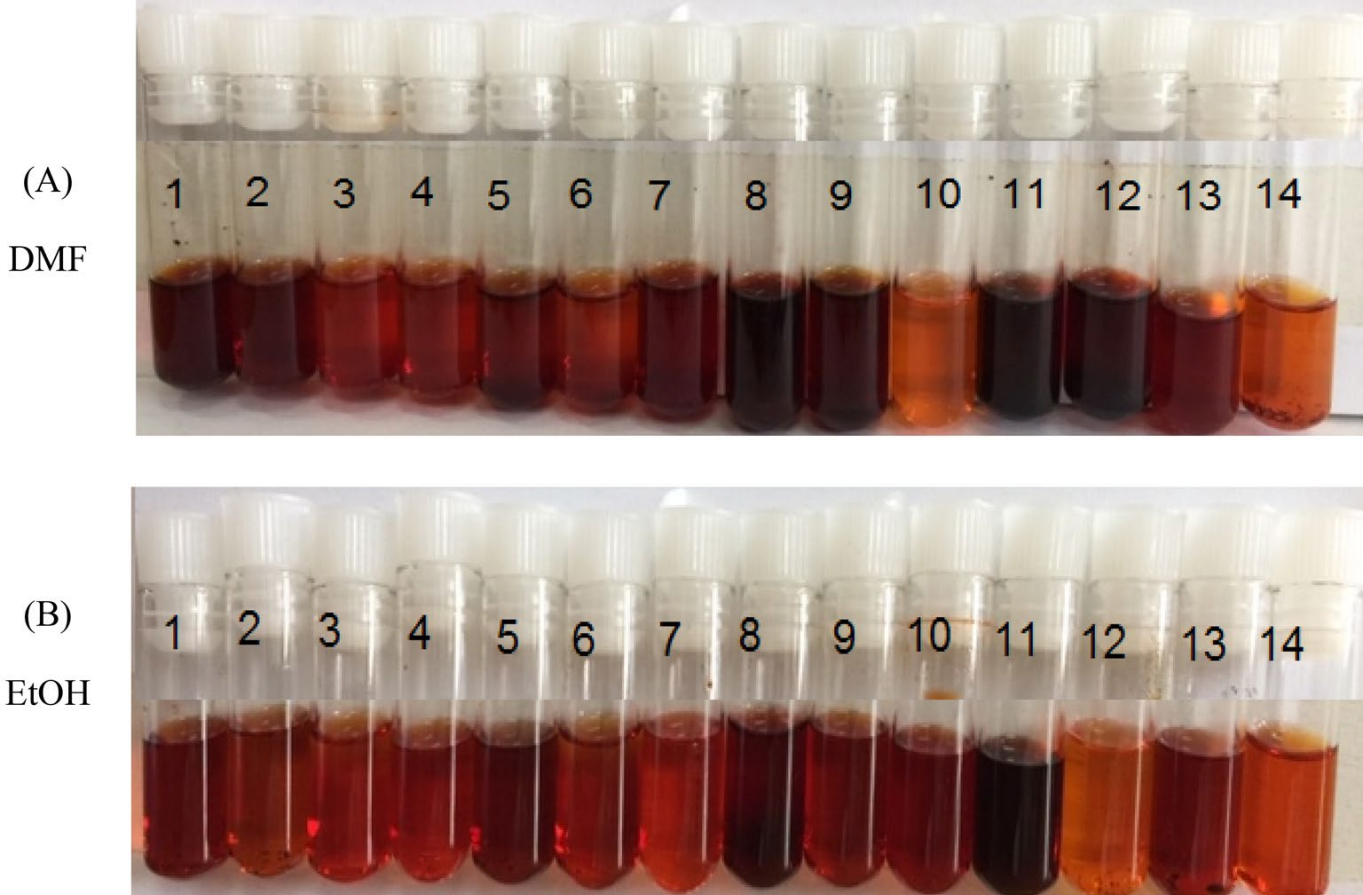
Colors of the solutions for dyes **1–14** under room temperature: in (**A**) DMF and (**B**) EtOH.

The visual assessment of a dye solution can provide insights into its concentration, composition, and stability. Figure 6 illustrates the colors produced by dyes **1–14** when dissolved in DMF or ethanol at room temperature. Almost all dyes in DMF exhibit the same color with an exception for the substituent X = 2–CH_3_, X = 3–CH_3_, X = 2–OCH_3_, X = 4–OCH_3_, which have a rich, reddish-brown hue. In ethanol, most dyes exhibit the same color except X = 2–CH_3_ and X = 2–OCH_3,_ whose hues are more vibrant than those of the others.

### Dyeing process on polyester (PE) fabrics:

**Figure 7:**
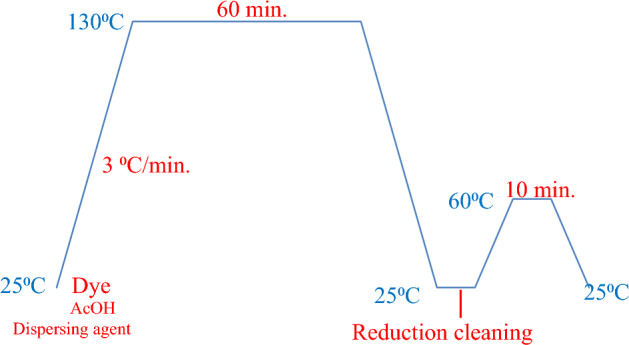
One bath dyeing profile of polyester fabrics with dispersed dyes** 1–14.**

The dyeing process of disperse azo dyes on polyester fabrics is a complex procedure that demands meticulous attention to detail to achieve the desired color and quality, Fig. 7.

**Figure 8:**
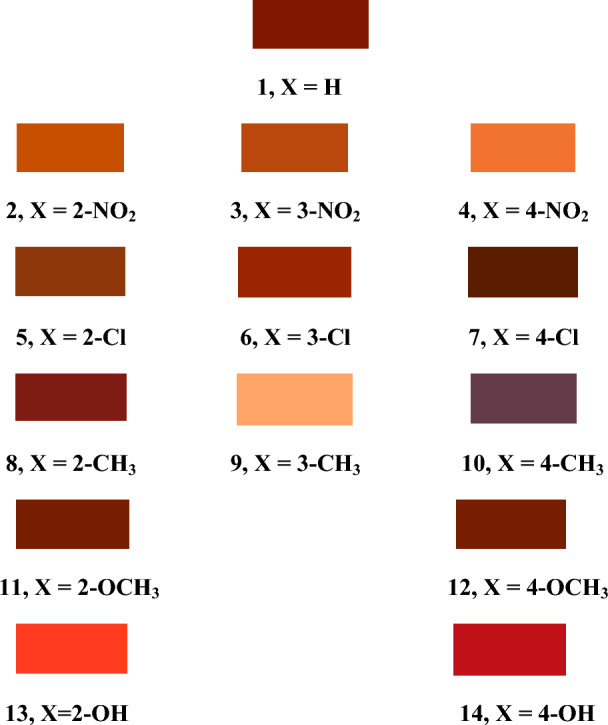
Color shades of polyester dyed samples by dyes **1–14**.

The polyester fabrics were dyed with the newly synthesized disperse dyes **1–14** using a high-temperature pressure technique (130 °C) at a shade of 2%, Fig. 8. The fastness properties of the dyed polyester fabrics were evaluated as shown in Table 3.

**Table 3:** Fastness properties of polyester fabrics dyed with disperse azo dyes **1–14**.

Dye no	X-		Shade Color name	Wash* fastness	Perspiration fastness** (cotton)		Perspiration** fastness (polyester)		Scorch fastness***(180 °C)		Light**** fastness (48 h)
					Acidic	Alkaline	Acidic	Alkaline	Cotton	Polyester	
1	4–H		Apple of my eye	1	4	4	5	2–3	3	2	6–7
2	2–NO_2_		Vermillion orange	3	4	4	4	2–3	5	5	6–7
3	3–NO_2_		Orange Popsicle	1–2	3	3–4	2–3	3–4	4–5	4–5	6–7
4	4–NO_2_		Orange Glory	4	4	4	4	4	5	4–5	6–7
5	2–Cl		Brown	4	2–3	4	2	3	3–4	2–3	3
6	3–Cl		Cancun coral	2–3	3	4	3	3	3–4	2	7–8
7	4–Cl		Apple of my eye	4	4	5	4	4	5	3–4	4
8	2–CH_3_		Zydeco red	1	2–3	2	2	3	2–3	1–2	5–6
9	3–CH_3_		Peach amber	4–5	2–3	3–4	4	4	5	5	3
10	4–CH_3_		Fun purple	4–5	4	3–4	4	3–4	5	5	3
11	2–OCH_3_		Ultimate red	1–2	3	4	2–3	4	3	2–3	5–6
12	4–OCH_3_		Smoldring orange	4–5	2–3	3–4	2	4	4–5	3	4–5
13	2–OH		Living coral	3	3–4	4	4	4	3–4	3	3
14	4–OH		Bright rose	1–2	3–4	3	3–4	3–4	4–5	3	6–7

*AATCC16 (ISO 105 C06); **AATCC15 (ISO 105 E04); ***AATCC13 (ISO 105 X11); ****AATCC 16 (ISO 105 B02).

The observed variations in color shades among the dyed polyester samples can be attributed to the nature and position of the auxochrome X in the arylazo moiety, which penetrated inside the fibers. It is observed that there is a small change in the shade for the same substituent at different positions of the substituent in the phenyl moiety except for methyl substituent, which resulted in significantly different shades depending on its position. On the other hand, the change-like substituents showed observable changes in the color shade of the fiber. Also, the substituent at position 4- showed a more pronounced change in color shade compared to those at positions 2- and 3-.

#### Color fastness

Color fastness generally means that the color on a dyed fiber withstands the effects of washing, rubbing, light exposure, and other environmental factors. The level of color fastness depends on various factors such as the type of dyestuff used, the dyeing technique, the fiber type, and the conditions to which the fiber is subjected. High color fastness is important for ensuring the longevity of dyed fabrics and their ability to resist fading and discoloration.

##### Fastness to Washing.

The dyed polyester fabrics by dyes **4, 5, 7, 9, 10**, and **12** exhibit excellent fastness to fading and washing, as measured by the international Geometric Greyscale^42,43^. Dyes **2, 6**, and **13** exhibits moderate fastness to washing with grade 3 in greyscale. The reminder dyes show poor fastness to washing. Reasons for this include insufficient diffusion of dye molecules into the textiles and the dye-fiber affinity.

##### Fastness to perspiration: (Acid and Alkaline)

The high ratings for perspiration fastness for most dyed fabrics, regardless of pH conditions, suggest that the pH level does not affect the dyed samples' sensitivity. This could be attributed to the inherent stability of the dyes towards degradation under both acidic and basic conditions.

##### Fastness to scorch fastness (180 °C)

One of the most critical prerequisites for dyed polyester textiles is their scorch fastness, which refers to their ability to withstand heat treatment and the presence of a dispersion agent needed for the migration of dye molecules to polyester fibers. Generally, cotton samples exhibit better scorch fastness than polyester ones. Among the synthesized dyes, **2, 4, 9**, and **10** demonstrate excellent fastness to hot pressing at 180 °C and excellent photofading resistance.

##### Fastness to Light

Fastness to light is the ability of a substance to resist fading or color change when exposed to light. It is commonly measured in textile and dye industries, as well as in printing and painting. This property is essential in products that are meant to be used or displayed outdoors, such as outdoor furniture, fabrics, and signage. The measurement of fastness to light is usually done by exposing the sample to a specified amount of light and comparing the color change to a standardized scale. The scale ranges from 1 to 8, with 1 being the least resistant to light and 8 being the most resistant. Higher fastness to light rating means that the material retains its color and appearance longer, making it more durable and long-lasting. Dyed sample by dyes **1–4, 6** and **14** generally exhibited acceptable light fastness, with ratings ranging from 6 to 7 on the international Geometric Grey Scale^42,43^.

In general, materials with electron-donating groups can be more susceptible to photodegradation, as they can absorb light more readily and undergo photochemical reactions. It has been observed that electron-accepting groups can enhance the light fastness of the azo dyes. This observation is consistent with the notion that azo dyes containing electron-withdrawing substituents on their diazo components exhibit reduced susceptibility to photofading^44^.

#### Dye exhaustion

The exhaustion efficiency (E%) values of disperse azo dyes **1–14** on polyester samples are shown in Table 4. It shows that the exhaustion efficiencies values are relatively correlated with the nature and position of the substituent in the aryl moiety. Disperse dye **1** has the highest dye E%, while dye **4** has the lowest E% (43.75%) that appeared from the photofading of the dyed fabrics. The order of E% is 4–H > 3–Cl > 3–NO_2_, 4–Cl, 4–CH_3_ > 2–NO_2_, 3–CH_3_ > 4–OH > 2–OH > 2–CH_3_ > 2–Cl > 2–OCH_3_, 4–OCH_3_ > 4–NO_2_. This order indicates that the nature of the substituent has a great effect on exhaustion values. Both the fabric's structure and the nature and location of the substituent X- may have a role in this. Substituents may also aid and affect the dye's ability to penetrate and infiltrate the cloth to a desired depth.

**Table 4:** Dye exhaustion (E%) of polyester fabrics dyed with azo dyes **1–14.**

Dye	1	2	3	4	5	6	7	8	9	10	11	12	13	14
X-	4–H	2–NO_2_	3–NO_2_	4–NO_2_	2–Cl	3–Cl	4–Cl	2–CH_3_	3–CH_3_	4–CH_3_	2–OCH_3_	4–OCH_3_	2–OH	4–OH
*E%*	99.0	95.0	97.5	43.8	87.5	98.8	97.5	88.0	95.0	97.5	62.5	62.5	88.8	90.0

#### Color measurement

The effect of the nature of the various substituents on dyeing behavior, color hue, and depth was analyzed in this study, relying on spectral data from the dyed materials. To evaluate the optical properties of the samples, the Kubelka–Munk function f(R) was employed, which describes the absorption "K" and scattering "S" of light. The absorption "K" is primarily determined by the dyestuffs. At the same time, the substrate influences the scattering " S. "The reflectance (R) of a thick, opaque sample with a constant value of "K" and "S" can be calculated using the Kubelka–Munk theory based on the wavelength of light, Eq. (1).

1 $${\text{K}}/{\text{S}} = \left( {{1} - {\text{R}}} \right)^{{2}} /{\text{2R}}$$

The color properties of the dyed samples were evaluated using various methods. The K/S value at λ_max_ was used to determine the color depth, while the psychometric coordinates (L*, a*, b*) were obtained to illustrate the color hues.

In the calculation of color differences (ΔL*, ΔC*, ΔH*, and ΔE), the parent dyestuff **1** (X = H) was used as the standard^45,46^. The CIE-LAB technique was used to obtain these values, which are presented in Table 5, where ΔL*: is the lightness difference, ΔC*: is the chroma difference, ΔH*: is the hue difference, and ΔE: is the total color difference. A negative ΔL* value indicated that the dyed fiber was darker than the standard, while a positive value indicated that the dyed fiber was lighter. The nature and position of the substituents on the dye molecules influenced the color properties of the dyed samples.

**Table 5:** Reflectance, color strength, and colorimetric colors CILAB of dyed polyester fabrics by azo dyes **1–14.**

Code	X	L*	a*	b*	c*	h*	ΔL*	ΔC*	ΔH*	ΔE	R	K/S
1	4–H	28.6	27.7	21.9	35.3	38.4	–	–	–	–	1.5	28.2
2	2–NO_2_	46.3	31.4	30.6	43.8	44.3	17.8	8.5	5.9	20.6	4.8	9.7
3	3–NO_2_	44.6	30.3	35.9	46.9	49.8	16.0	11.6	11.5	22.9	3.3	14.9
4	4–NO_2_	52.0	31.0	26.6	40.9	40.7	23.5	5.5	2.3	24.2	7.8	5.8
5	2–Cl	38.9	23.4	17.7	29.3	37.1	10.4	− 6.0	− 1.2	12.1	3.3	9.9
6	3–Cl	37.1	31.3	31.6	44.5	45.3	8.6	9.1	6.9	14.3	0.5	24.5
7	4–Cl	31.5	26.9	18.8	32.8	35.0	3.0	− 2.5	− 3.3	5.1	0.8	20.9
8	2–CH_3_	27.1	30.5	21.3	37.2	34.9	− 1.4	1.9	− 3.5	4.2	1.4	32.4
9	3–CH_3_	55.1	20.7	14.9	25.4	35.8	26.5	− 9.9	− 2.6	28.4	14.9	2.4
10	4–CH_3_	29.7	9.1	1.5	9.3	9.5	1.2	− 26.1	− 28.9	38.9	5.3	8.6
11	2–OCH_3_	28.2	27.8	11.2	29.9	22.0	− 0.4	− 5.4	− 16.3	17.2	1.9	22.0
12	4–OCH_3_	38.3	26.0	17.9	31.6	34.7	9.7	− 3.6	− 3.7	11.0	4.3	11.5
13	2–OH	43.8	40.2	20.8	45.2	27.4	15.3	9.9	− 10.9	21.3	4.9	10.2
14	4–OH	37.5	46.5	22.5	51.6	25.8	8.9	16.3	− 12.5	22.4	3.7	18.1

Table 5 shows that the values of K/S of compounds **1**–**14** vary from 2.37 to 32.43. The introduction of electron-withdrawing and electron-donating groups in the aryl group has lower K/S values than that of parent **1,** except dye **8** (X = 2–CH_3_) has the higher K/S than dye **1,** Table 5. It was found that all dyed fabrics **1–14** have a positive value of a* and b*, which indicate a shift in the color hues of the dye to a reddish direction on the red-green axis and to a yellowish direction on the yellow-blue axis, respectively. Negative hue angle (H*) values indicate a shift towards a reddish hue, whereas positive values indicate that the color shifted to yellowish, represented by all synthesized dyes **1–14**. The results in Table 4 indicate that the most effective dye on the brightness value (L*) of the PE samples is **9**. All dyes with (+ ve ΔC) values are brighter than the parent dye **1**. The negative sign of ΔC* for dyes **5, 7**, and **9–12** indicates that the dyed fiber becomes duller than the standard **1**, but a positive sign as in dyes **2–4, 6, 8**, and **13–14** indicates that the dyed fiber become brighter than the standard. The negative sign of ΔH* in dyes **5** and **7–14** indicates that the color is directed to red color while a positive sign as in **2–4** and **6** indicates that the color is directed to yellowish. The results of the color difference (ΔE), which represents the complete changes in the three color components, are given in Table 4. They confirm that most tested dyes caused real color changes for the dyed PE samples. The color difference value (ΔE) of the dyed PE samples was calculated in relation to unsubstituted dye (X = H).

## Conclusion

The synthesized disperse azo dyes **1–14** existed in the azo form in the solid state and the hydrazone form in the DMSO solution. Azo-hydrazone equilibrium persisted in dimethyl formamide that appeared from UV–Vis spectroscopy, indicating the presence of both transitions for the azo and hydrazo groups. The DFT studies estimated λ_max_ of the titled dyes and agreed with the experimental absorption data. The change in the aniline moiety substituents affects dyed polyester fabrics' color shading. Fabrics dyed by disperse dye **7** had the deepest color shading, while dye **9** gave the lightest color shade. Dyes **4, 5, 7, 9, 10**, and **12** have very good fastness to washing, while dyes **2, 4, 9,** and **10** exhibits excellent fastness to hot pressing at 180 °C and excellent photofading resistance. The order of E% is 4–H > 3–Cl > 3–NO_2_, 4–Cl, 4–CH_3_ > 2–NO_2_, 3–CH_3_ > 4–OH > 2–OH > 2–CH_3_ > 2–Cl > 2–OCH_3_, 4–OCH_3_ > 4–NO_2_. K/S of dyed polyester by disperse dyes **1**–**14** lie in the range of 2.37 to 32.43. Both electron-withdrawing and electron-donating groups, resulting in a lower K/S ratio than in parent 1.

## Experimental

### Materials and chemicals

2,3-Dihydroxynaphthalene (2,3-DHN), aniline, 2-, 3- and 4-nitroaniline, 2-, 3- and 4-chloroaniline, 2-, 3- and 4-methylaniline, 2- and 4-methoxy aniline, 2- and 4-hydroxy aniline, sodium hydroxide (NaOH), sodium carbonate (Na_2_CO_3_), and concentrated HCl, methanol, ethanol, dimethyl formamide (DMF), Silica gel. were purchased from Sigma-Aldrich and were used without further purification. Liquid chemicals were used after distillation. For purification purposes, methanol: ethyl acetate eluent is used, and the purified dyes were dried by standard method.

### Instruments and apparatus

The melting point was determined using the MEL-TEMP II melting point apparatus with open glass capillaries. The infrared spectra were recorded by preparing potassium bromide (KBr) discs and analyzing them using a Perkin–Elemer FT-IR (Fourier-Transform Infrared Spectroscopy) instrument at the Faculty of Science at Alexandria University. For the NMR spectra, tetramethylsilane (TMS) was utilized as an internal standard, and the measurements were performed on a (JEOL) 500 MHz spectrophotometer at room temperature (~ 25 °C), NMR Unit of the Faculty of Science at Mansoura University. Elemental analyses were conducted at the Regional Centre for Mycology and Biotechnology et al.-Azhar University in Cairo, Egypt.

### General method for synthesis of dyes 1–14

Diazotizing primary aromatic amines and coupling with the corresponding 2,3-DHN synthesized the investigated mono-azo dye **1–14** derivatives.

In a 250 mL capacity conical flask, a mixture of aniline derivatives (0.008 mol), 7 mL conc. HC1 and 14 mL water were stirred to clear the solution and kept cooling at 0° to − 5° C in an ice bath. Add 5 g sodium nitrite into aniline hydrochloride solution dropwise with constant stirring.

Add the prepared diazonium salt solution slowly to a well-cooled mixture of the coupler, namely, 2,3-dihydroxynaphthalene (0.006 mol) dissolved in 15 mL H_2_O and 0.3 g sodium hydroxide in 15 mL of water (keep the mixture alkaline). TLC monitored the progress of the reaction, and then crude dyes were filtered and washed with hot water several times. The crude was purified by flash column using ethyl acetate: hexane mixture (1:6) as eluent.

#### 3-Hydroxy-1-(2-phenylhydrazineylidene)naphthalen-2(1*H*)-one 1

Reddish brown crystal, 1.2 g (68.6%) yield; m.p.200 °C (decompose). UV: λ_max_ (DMF): 470 nm, and Ɛ_max_ 15,000 mol^−1^dm^3^cm^−1^. IR (KBr): ῡ 3433 (OH), 3066 (SP^2^ = C–H), 1567 (N=N) cm^−1^. ^1^H NMR (400 MHz, *d6*-DMSO): δ 16.09 (s, 1H, OH), 9.68 (s, 1H, NH), 8.28 (d, *J* = 7.6 Hz, 1H, Ar–H), 7.73 (d, *J* = 7.6 Hz, 2H, Ar–H), 7.58 –7.46 (m, 3H, Ar–H), 7.34 (m, 3H, Ar–H) and 7.14 (s, 1H, Ar–H) ppm. ^13^C NMR (101 MHz, *d6*-DMSO): δ 151.02, 150.40, 149.47, 147.39, 147.09, 146.79, 146.41, 131.02, 128.28, 127.36, 125.71, 122.77, 115.54, 115.24, 110.96 and 110.28 ppm. C_16_H_12_N_2_O_2_ requires: C, 72.71; H,4.59; N, 10.61%, Found: C, 72.90; H, 4.43; N, 10.66%

#### 3-Hydroxy-1-(2-(2-nitrophenyl)hydrazineylidene)naphthalene-2(1*H*)-one 2

Extra orange crystal, 1 g (51.8%) yield; m.p. 240 °C. UV: λ_max_ (DMF) 435 nm, and Ɛ_max_ 21,498 mol^−1^dm^3^cm^−1^. IR (KBr): IR (KBr): ῡ 3205 (OH), 3099 (SP^2^ = C–H), 1574 (N=N), 1525 (NO_2_ asymmetric) and 1317 (NO_2_ symmetric) cm^−1^. ^1^H NMR (400 MHz, *d6*-DMSO): δ 13.95 (s, 1H, OH), 9.74 (s, 1H, NH) and 7.65–8.40 (m, 9H, 9Ar-H) ppm. C_16_H_11_N_3_O_4_ requires C, 62.12; H, 3.60; N, 13.60%, found: C, 60.21; H, 3.57; N,13.72.

#### 3-Hydroxy-1-(2-(3-nitrophenyl)hydrazineylidene)naphthalen-2(1*H*)-one 3

Extra orange crystal, 1.2 g (63.2%) yield; m.p.149 °C. UV: λ_max_ (DMF) 465 nm, and Ɛ_max_ 7102 mol^−1^dm^3^cm^−1^. IR (KBr): IR (KBr): ῡ 3270 (OH), 3070 (SP^2^ = C–H), 1572 (N=N), 1529 (NO_2_ asymmetric) and 1347 (NO_2_ symmetric) cm^−1^. ^1^H NMR (400 MHz, *d6*-DMSO): δ 15.63 (s, 1H, OH), 9.74 (s, 1H, NH), 8.56 (s, 1H, Ar–H), 8.27 (d, *J* = 7.3 Hz, 1H, Ar–H), 8.21 (d, *J* = 9.2 Hz, 1H, Ar–H), 8.08 (d, *J* = 9.7 Hz, 1H, Ar–H), 7.78 (t, *J* = 8.1 Hz, 1H, Ar–H), 7.51 (s, 1H, Ar–H), 7.39 (d, *J* = 9.5 Hz, 2H, Ar–H) and 7.11 (s, 1H, Ar–H) ppm.^13^C NMR (101 MHz, *d6*-DMSO): δ 173.66, 149.44, 149.32, 144.32, 131.61, 130.99, 129.52, 128.81, 127.69, 127.64, 126.88, 123.65, 121.99, 120.30, 117.69 and 111.82 ppm. C_16_H_11_N_3_O_4_ requires C, 62.12; H, 3.60; N, 13.60%, found: C, 62.01; H, 3.65; N, 13**.**55.

#### 3-Hydroxy-1-(2-(4-nitrophenyl)hydrazineylidene)naphthalen-2(1H)-one 4

Extra orange crystal, 1.3 g (67.4%) yields; m.p.159–160 °C. UV: λ_max_ (DMF) 470 nm, and Ɛ_max_ 13,750 mol^−1^dm^3^cm^−1^. IR (KBr): IR (KBr): ῡ 3203 (OH), 3076 (SP^2^ = C–H), 1594 (–N=N), 1509 (NO_2_ asymmetric) and 1337 (NO_2_ symmetric) cm^−1^. ^1^H NMR (400 MHz, *d6*-DMSO): δ 15.41 (s, 1H, OH), 9.52 (s, 1H, NH) and 8.28–7.28 (m, 9H, 9Ar-H) ppm. ^13^C NMR (101 MHz, *d6*-DMSO) δ 150.92, 149.47, 147.68, 147.33, 146.62, 131.72, 131.02, 129.24, 128.28, 127.48, 123.36, 117.48, 111.58 and 109.98 ppm. C_16_H_11_N_3_O_4_ requires C, 62.12; H, 3.60; N, 13.60% found: C, 61.98; H, 3.69; N,13**.**56.

#### 1-(2-(2-Chlorophenyl)hydrazineylidene)-3-hydroxynaphthalen-2(1*H*)-one 5

Extra orange crystal, 1.3 g (68.4%) yields; m.p.240 °C (Decompose). UV: λ_max_ (DMF) 470 nm, and Ɛ_max_ 4913.5 mol^−1^dm^3^cm^−1^. IR(KBr): ῡ 3437 (OH), 3099 (SP^2^ = C–H) and 1592 (N=N) cm^−1^. ^1^H NMR (400 MHz, *d6*-DMSO): δ 14.34 (s, 1H, OH), 9.71 (s, 1H, NH), 7.64 (d, *J* = 7.8 Hz, 1H, Ar–H), 7.60 –7.47 (m, 2H, Ar–H), 7.45–7.34 (m, 3H, Ar–H), 7.29 (t, *J* = 12.2 Hz, 1H, Ar–H), 7.13 (s, 1H, Ar–H) and 6.82 (s, 1H, Ar–H) ppm.^13^C NMR (101 MHz, *d6*-DMSO): δ 174.05, 168.41, 149.64, 139.24, 131.28, 130.92, 130.58, 129.54, 129.25, 128.27, 127.23, 126.60, 121.86, 121.57, 117.56 and 116.90 ppm. C_16_H_11_N_2_O_2_Cl requires C, 64.32; H, 3.72; N, 9.38%. Found: C, 64.29; H, 3.78; N, 9.31.

#### 1-(2-(3-Chlorophenyl)hydrazineylidene)-3-hydroxynaphthalen-2(1*H*)-one 6

Flame scarlet crystal, 1.0 g (53.8%) yields; m.p. 160 °C. UV: λ_max_ (DMF) 465 nm, and Ɛ_max_ 11,675 mol^1^dm^3^cm^−1^. IR (KBr): ῡ 3444 (OH), 3073 (= C–H) and 1592 (N=N) cm^−1^. ^1^H NMR (400 MHz, CDCl_3_): δ 15.89 (s, 1H, OH), 9.11 (s, 1H, NH), 8.30 (d, *J* = 8.7 Hz, 1H, Ar–H), 7.70 (s, 1H, Ar–H), 7.51–7.32 (m, 5H, Ar–H), 7.23 (d, *J* = 7.6 Hz, 1H, Ar–H) and 7.09 (s, 1H, Ar–H) ppm. ^13^C NMR (101 MHz, CDCl_3_): δ 172.58, 147.78, 143.13, 135.83, 130.73, 130.14, 129.88, 128.74, 128.57, 128.49, 128.37, 127.95, 127.59, 127.18, 126.83 and 126.22 ppm. C_16_H_11_N_2_O_2_Cl requires 64.32; H, 3.72; N, 9.38%. Found: C, 64.28; H, 3.65; N, 9.43.

#### 1-(2-(4-Chlorophenyl)hydrazineylidene)-3-hydroxynaphthalen-2(1*H*)-one 7

Flame scarlet crystal, 1.0 g (53.7%) yield; m.p.180 °C. UV: λ_max_ (DMF) 480 nm, and Ɛ_max_ 15,862.75 mol^−1^dm^3^cm^−1^. IR (KBr): ῡ 3354 (OH), 3095 (SP^2^ = C–H) and 1566 (N=N) cm^−1^. ^1^H NMR (400 MHz, CDCl_3_): δ 16.08 (s, 1H, OH), 8.33 (d, *J* = 7.8 Hz, 1H, Ar–H), 7.61 (d, *J* = 8.9 Hz, 2H, Ar–H), 7.45 (m, 5H, Ar–H) and 7.12 (s, 1H, Ar–H) ppm. ^13^C NMR (101 MHz, *d6*-DMSO): δ 171.50, 149.39, 142.11, 141.82, 130.80, 130.21, 130.01, 129.10, 128.82, 127.75, 127.19, 126.57, 119.51 and 118.24 ppm. C_16_H_11_N_2_O_2_Cl requires 64.32; H, 3.72; N, 9.38%. Found: C, 64.41; H, 3.66; N, 9.45.

#### 3-Hydroxy-1-(2-(2-tolyl)hydrazineylidene)naphthalen-2(1*H*)-one 8

Zydeco red crystal, 1.3 g (75.1%) yield; m.p.170 °C (decompose). UV: λ_max_ (DMF) 480 nm, and Ɛ_max_ 18,967 mol^−1^dm^3^cm^−1^. IR (KBr): ῡ 3388 (OH), 3030 (SP^2^ = C–H), 2974 (SP^3^ –C–H) and 1561 (N=N) cm^−1^. ^1^H NMR (400 MHz, CDCl_3_): δ 14.48 (s, 1H, OH), 8.37 (d, *J* = 6.6 Hz, 1H, Ar–H), 8.08 (d, *J* = 7.4 Hz, 2H, Ar–H), 7.53–7.33 (m, 3H, Ar–H), 7.33–6.98 (m, 3H, Ar–H) and 2.52 (s, 3H, CH_3_) ppm. ^13^C NMR (101 MHz, CDCl_3_): δ 171.17, 147.96, 140.18, 137.23, 131.14, 129.99, 128.94, 128.09, 127.67, 127.42, 126.61, 126.52, 122.02, 121.38, 115.87, 115.78 and 17.01 ppm. C_17_H_14_N_2_O_2_ requires C, 73.36; H, 5.08; N, 10.07; % found: C, 73.41; H, 5.15; N, 10.17.

#### 3-Hydroxy-1-(2-(3-tolyl)hydrazineylidene)naphthalen-2(1*H*)-one 9

Zydeco red crystal,1.4 g (80.5%) yield; m.p.164 °C (decompose). UV: λ_max_ (DMF) 475 nm, and Ɛ_max_ 20,407 mol^−1^dm^3^cm^−1^. IR (KBr): ῡ 3350 (OH), 3099 (SP^2^ = C–H), 2922 (SP^3^ –C–H) and 1567 (N=N) cm^−1^. ^1^H NMR (400 MHz, CDCl_3_): δ 14.22 (s, 1H, OH), 9.21 (s, 1H, NH), 8.36 (d, *J* = 7.8 Hz, 1H, Ar–H), 7.57– 7.31 (m, 6H, Ar–H), 7.11 (s, 2H, Ar–H) and 2.47 (s, 3H, CH_3_) ppm. ^13^C NMR (101 MHz, CDCl_3_): δ 171.46, 147.97, 141.86, 139.94, 129.63, 129.28, 129.02, 128.10, 127.71, 127.45, 126.61, 126.53, 121.40, 117.75, 115.90, 114.59 and 21.57 ppm. C_17_H_14_N_2_O_2_ requires 73.36; H, 5.08; N, 10.07% found: C, 73.28; H, 5.03; N, 10.17.

#### 3-Hydroxy-1-(2-(4-tolyl)hydrazineylidene)naphthalen-2(1*H*)-one 10

Zydeco red crystal, 1.1 g (72.4%) yield; m.p.210 °C (Decompose). UV: λ_max_ (DMF) 525 nm, and Ɛ_max_ 11,871 mol^1^dm^3^cm^−1^, UV: λ_max_ (DMF) 375 nm, and Ɛ_max_ 15,979 mol^1^dm^3^cm^−1^. IR (KBr): ῡ 3448 (OH), 3171 (SP^2^ = C–H), 2919 (SP^3^ –C–H) and 1580 (N=N) cm^−1^.^1^H NMR (400 MHz, *d6*-DMSO): δ 14.70 (s, 1H, OH), 9.61 (s, IH, NH), 8.75 (s, 1H, Ar–H), 8.31–8.08 (m, 1H, Ar–H), 7.76–7.48 (m, 4H, Ar–H), 7.47–7.22 (m, 3H, Ar–H) and 2.34 (s, 3H, CH_3_) ppm. ^13^C NMR (101 MHz, *d6*-DMSO): δ 174.20, 162.93, 140.29, 135.37, 130.98, 130.91, 130.70, 129.07, 128.74, 127.36, 123.23, 116.84, 116.67, 103.07 and 20.91 ppm. C_17_H_14_O_2_N_2_ requires 73.36; H, 5.08; N, 10.07%; found: C, 73.32%; H, 5.14%; N, 10.27.

#### 3-Hydroxy-1-(2-(2-methoxyphenyl)hydrazineylidene)naphthalen-2(1*H*)-one 11

Living coral, 1.2 g (67.4%) yield; m.p.200 °C. UV: λ_max_ (DMF) 480 nm, and Ɛ_max_ 14,473 mol^1^dm^3^cm^−1^. IR (KBr): ῡ 3374 (OH), 3072 (SP^2^ = C–H), 2932 (SP^3^ –C–H), 1561 (N=N), cm^−1^. ^1^H NMR (400 MHz, *d6*-DMSO): δ 15.49 (s, 1H, OH), 9.68 (s, 1H, NH), 8.32 (d, *J* = 7.2 Hz, 1H, Ar–H), 8.07 (d, *J* = 7.8 Hz, 1H, Ar–H), 7.50 (d, *J* = 7.6 Hz, 1H, Ar–H), 7.42–7.21 (m, 4H, Ar–H), 7.22–7.13 (m, 1H, Ar–H), 7.10 (s, 1H, Ar–H) and 4.01 (s, 3H, OCH_3_) ppm. ^13^C NMR (101 MHz, *d6*-DMSO): δ 174.34, 172.39, 150.02, 149.03, 131.65, 130.68, 129.02, 128.04, 127.37, 126.70, 123.43, 122.46, 121.50, 116.84, 115.85, 112.91 and 57.36 ppm. C_17_H_14_O_3_N_2_ requires C, 69.37; H, 4.80; N,9.52%; found: C, 69.25; H, 4.74; N, 9.55.

#### 3-Hydroxy-1-(2-(4-methoxyphenyl)hydrazineylidene)naphthalen-2(1*H*)-one 12

Living coral, 1.0 g (54.3%) yield; m.p.210–212 °C. UV: λ_max_ (DMF) 408 nm, and Ɛ_max_ 5629 mol^−1^dm^3^cm^−1^. IR (KBr): ῡ 3417 (OH), 3085 (SP^2^ = C–H), 2927 (SP^3^ –C–H) and 1601 (N=N) cm^−1^. ^1^H NMR (400 MHz, *d6*-DMSO): δ 15.55 (s, 1H, OH), 9.68 (s, 1H, NH), 8.71–6.65 (m, 9H, Ar–H), 3.86 (s, 1H, OCH_3_). C_17_H_14_O_3_N_2_ requires C, 69.37; H, 4.80; N,9.52% found: C, 69.41; H, 4.84; N, 9.63.

#### 3-Hydroxy-1-(2-(2-hydroxyphenyl)hydrazineylidene)naphthalen-2(1*H*)-one 13

Vibrant orange, 1.0 g (58.3%) yield; m.p.180 °C (decompose). UV: λ_max_ (DMF) 475 nm, and Ɛ_max_ 8231.75 mol^−1^dm^3^cm^−1^. IR (KBr): ῡ 3348 (OH), 3075 (SP^2^ = C–H) and 1517 (N=N) cm^−1^. ^1^H NMR (400 MHz, *d6*-DMSO): δ 14.34 (s, 1H, OH), 9.62 (s, 1H, NH), 8.80 (s, 1H, OH), 7.44–7.34 (m, 4H, Ar–H), 7.30 (t, *J* = 7.4 Hz, 2H, Ar–H) and 6.82 (s, 2H, Ar–H) ppm.^13^C NMR (101 MHz, *d6*-DMSO): δ 149.47, 131.69, 131.02, 129.29, 128.77, 128.28, 127.48, 126.40, 126.00, 125.48, 125.27, 124.40, 123.32, 117.57, 111.54 and 109.98 ppm. C_16_H_12_N_2_O_3_ requires C, 68.56; H, 3.96; N, 9.99%. Found: C, 68.65; H, 3.91; N, 10.03.

#### 3-Hydroxy-1-(2-(4-hydroxyphenyl)hydrazineylidene)naphthalen-2(1*H*)-one 14

Vibrant orange, 0.8 g (46.9%) yield; m.p.120 °C. UV: λ_max_ (DMF) 480 nm, and Ɛ_max_ 18,283 mol^−1^dm^3^cm^−1^. IR (KBr): ῡ 3380 (OH), 3062 (SP^2^ = C–H) and 1596 (N=N) cm^−1^. ^1^H NMR (400 MHz, *d6*-DMSO): δ 16.06 (s, 1H, OH), 10.13 (s, 1H, OH), 9.67 (s, 1H, NH), 8.45 (d, *J* = 7.9 Hz, 1H, Ar–H), 7.78 (d, *J* = 8.7 Hz, 2H, Ar–H), 7.60 (d, *J* = 7.6 Hz, 1H, Ar–H), 7.37 (m, 2H, Ar–H), 7.21 (s, 1H, Ar–H), 6.98 (d, *J* = 8.7 Hz, 2H, Ar–H). ^13^C NMR (101 MHz, *d6*-DMSO): δ 159.66, 159.14, 148.49, 138.37, 129.15, 128.41, 128.30, 127.12, 125.78, 123.32, 121.76, 121.15, 117.00 and 115.62 ppm. C_16_H_12_N_2_O_3_ requires C, 68.56; H, 3.96; N, 9.99%. Found: C, 68.51; H, 3.89; N, 10.03.

### Dyeing process

The advent of disperse dyes, which are insoluble in water and are applied to synthetic fibers from aqueous dispersions, was a significant breakthrough in the coloration of synthetic fibers. These dyes are produced by grinding them in the presence of dispersing agents to create microscopically fine particles, which are then pan-dried to obtain a readily dispersible solid. These solids, which partition into the fiber from low dye bath concentrations, can then dye the hydrophobic fibers. In this study, synthesized disperse dyes **1–14** were applied to the polyester fabric at 2% shade using a high-temperature pressure technique at 130 °C.

### Instruments used in fastness experiments

Various experiments were performed using different instruments in accordance with the testing methods outlined above. The Light Fastness Tester TF421 was utilized to determine the light fastness values of the polyester samples, while the wash fastness values were tested using the Launder Meter TF418. The Perspirometer TF416A was used to test the perspiration fastness values of the dyed polyester sample. The samples' color strength and intensity were detected using a Reflectance spectrophotometer.

### The experiment of color fastness properties test

ASTM (American Society for Testing and Materials) and AATCC (American Association of Textile Chemists and Colorists) developed some standards for testing the color fastness properties that were applied in this research for testing the samples^47^.

#### Fastness to washing.

The wash fastness properties of the fabric samples were tested using the ASTM D435-42 Standard with the Launder Meter TF418. A sample of dyed PET material was sewn between two white cotton fabrics, with equal lengths of 5 × 2.5, and the color was assessed using the worldwide grey scale (1 represents poor and 5 represents excellent).

#### Fastness to light.

The light-fastness properties of the fabric samples were tested using the AATCC TM16.1 Standard with the Light Fastness Tester TF421. The fabric was exposed to high-energy radiation “Q-SUN Xenon Test Chamber, QLAB, USA” in a “fade-o-meter” for 18–20 h (scale: 1 for poor and 8 for outstanding).

#### Fastness to perspiration

The perspiration fastness properties of the fabric samples were tested using the AATCC TM15 Standard with the Perspirometer TF416A.

### Testing of FTIR of dyed sample fabrics

FT-IR analysis was performed using an Agilent Cary 630 FTIR Instrument to identify the chromophores present in the dyestuffs of the sample fabrics. The dyestuffs were arranged by cutting them to size (3.5" × 1") with scissors and placing them in the apparatus clamp. Transmission spectroscopy was used to pass infrared radiation through the samples, detecting the degree of absorption and presence of chromophores.

### Color intensity and color strength testing method

#### Dye exhaustion

For all dyes **1–14**, to determine the extent of dye exhaustion, the dye concentration in the dyebath was measured before and after the dyeing process using a UV/Vis spectrophotometer (pg instruments T80 +) at the maximum wavelength (λ_max_) of the dye. The percentage of dye exhaustion (%E) was calculated using Eq. (2).

2 $$\%E=\frac{{C}_{1}-{C}_{2}}{{C}_{1}}*100$$

C_1_ and C_2_ are the dye concentrations in the dye bath before and after dyeing, respectively.

#### Reflection Spectra:

The reflectance values of the dyed fabrics were measured spectrophotometrically at the λ_max_. Reflection spectra were recorded for the polyester fiber samples in the visible range (400–700 nm) at the maximum wavelength of the dye used by a Jasco-UV/vis/NIR-spectrometer V-570 (1) Spectrophotometer. The samples' corresponding color strength (K/S) values were calculated using the Kubelka–Munk equation^48^.

### Color measurements

The colorimetric properties of dyed PET fabrics were obtained using a Hunter Lab Ultra Scan PRO (Reston, Virginia, USA) in terms of CIELab values (L*, a*, b*, c*, h°) using a standard illuminant D65 and 10° observer with specular radiation excluded on a Minolta CM-3600d visible spectrophotometer^49^.

According to this system, three basic tristimulus components of color, namely, hue (h°), chroma (C*) (also referred to as saturation), and Lightness (L*) (also referred to as luminance), were measured. The hue angle was measured from 0 to 360°. The values of the two coordinates, a* and b*, were also determined. L* represents the lightness or darkness of a color (L* = 100 for white and L* = 0 for black), whereas a* = red to green (+ a* = redder, − a* = greener), and b* = yellow to blue (+ b* = yellower, − b* = bluer), and where the two 'color' axes intersect = neutral gray.

The chroma (c*) and hue angle (h°) were measured using Eqs. (3 and 4), respectively.

3 $$Chroma {C}^{*}= \sqrt{{a}^{*2}+{b}^{*2}}$$

4 $$Hue angle {h}^{o}={tan}^{-1 }\left(\frac{{b}^{*}}{{a}^{*}}\right)$$

ΔH* stated color hue difference values. ΔL* stated the differences in the lightness values. The values of ΔL*, ΔC* and ΔH* are calculated by the following Eqs. (5–8):

5 $$\Delta {\text{L}}* = {\text{L}}*\left( {{\text{standard}}\;{\text{value}},{\text{ X}} = {\text{H}}} \right) - {\text{L}}*\left( {{\text{substituted}}\;{\text{value}}} \right)$$

6 $$\Delta {\text{C}}* = {\text{c}}*\left( {{\text{standard}}\;{\text{value}},{\text{ X}} = {\text{H}}} \right) - {\text{c}}*\left( {{\text{substituted}}\;{\text{value}}} \right)$$

7 $$\Delta {\text{H}}* = {\text{h}}*\left( {{\text{standard}}\;{\text{value}},{\text{ X}} = {\text{H}}} \right) - {\text{h}}*\left( {{\text{substituted}}\;{\text{value}}} \right)$$

Or

8 $$\Delta {\text{H}}*{\text{ab}} = [(\Delta {\text{E}}*{\text{ab}})^{{2}} - (\Delta {\text{L}}*)^{{2}} - (\Delta {\text{C}}*{\text{ab}})^{{2}} ]^{\raise.5ex\hbox{$\scriptstyle 1$}\kern-.1em/ \kern-.15em\lower.25ex\hbox{$\scriptstyle 2$} }$$

ΔE stated color difference values, and the use of the following Eq. (9) achieves it:

9 $$\Delta {\text{E}}*{\text{ab}} = [(\Delta {\text{L}}*)^{{2}} + (\Delta {\text{a}}*)^{{2}} + (\Delta {\text{b}}*)^{{2}} ]^{\raise.5ex\hbox{$\scriptstyle 1$}\kern-.1em/ \kern-.15em\lower.25ex\hbox{$\scriptstyle 2$} }$$

#### Computational methodology

All geometry optimizations were performed using the density functional theory (DFT) at the B3LYP functional^50^. The calculations were carried out using the GAUSSIAN 09 package. A full optimization was performed up to a higher basis set denoted by 6-31G(d,p) because this basis set and The maximal wavelengths (ʎ_max_) were systematically investigated using TD-DFT/B3LYP/6-31G(d,p) method, based on the optimized ground state (Supplementary file 1).

### Supplementary Information

Supplementary Information.

## Data Availability

The data used and analyzed during the current study are available from the corresponding authors upon reasonable request.
