# Supplementary material for: Synthesis, characterization, and application of novel aryldiazenyl disperse dyes on polyester fabrics
Source: Sci Rep. 2023 Dec 6;13:21554. doi: 10.1038/s41598-023-48368-y (PMC10700463; doi:10.1038/s41598-023-48368-y)
Supplement: Supplementary file 1 — Supplementary Information. [file 41598_2023_48368_MOESM1_ESM.docx]

**Synthesis, Characterization, and Application of Novel Aryldiazenyl Disperse Dyes on Polyester Fabrics**

Alaa Z. Omar ^a,*^, Asmaa M. Khamis ^b^, Ezzat A. Hamed ^a^, Samir K. El-Sadany ^a^, Elsayed M. Abdel Rehim ^b^, Mohamed E. Elba ^b^, Mohamed G. Mohamed ^a^ and Mohamed A. El-Atawy ^a,c^

^a^ Chemistry Department, Faculty of Science, Alexandria University, P.O. 426 Ibrahemia, Alexandria 21321, Egypt

^b^ Chemistry Department, Faculty of Science, Damanhour University, Damanhour, Egypt

^c^ Chemistry Department, Faculty of Science, Taibah University, Yanbu 46423 Saudi Arabia.

^*^ Correspondence: Alaazaki@alexu.edu.eg; Tel.: +201111361784, P.O. 426 Ibrahemia, Alexandria 21321, Egypt

|  |  |
| --- | --- |

UV-Visible absorption spectra of dyes **1-14** in DMF+ HCl

|  |  |
| --- | --- |

UV-Visible absorption spectra of dyes **1-14** in DMF+ NaOH


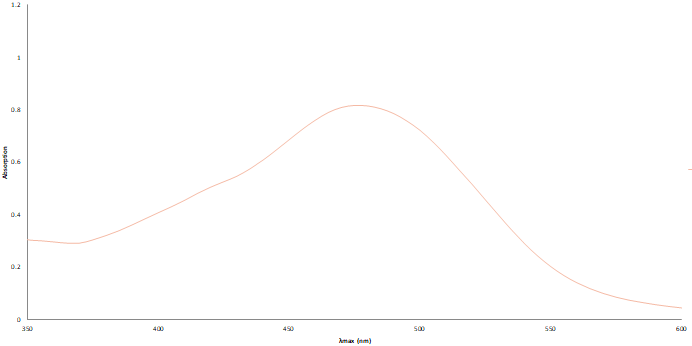


**UV spectrum of 3-hydroxy-1-(2-phenylhydrazineylidene)naphthalen-2(1*H*)-one 1**


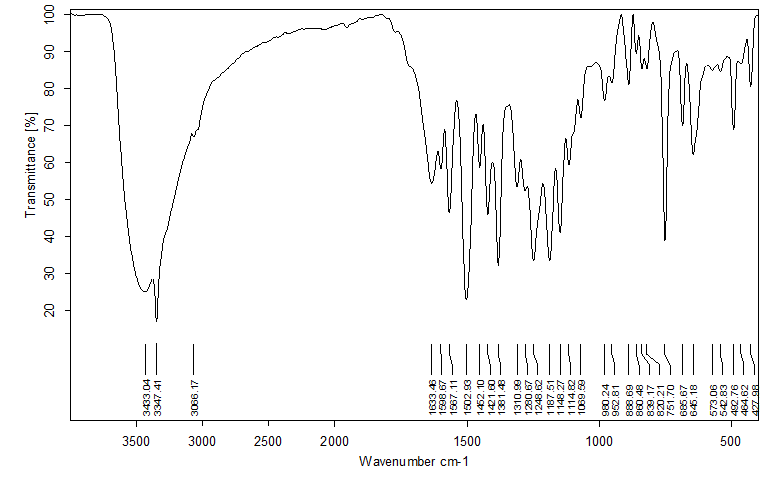


**IR spectrum of 3-hydroxy-1-(2-phenylhydrazineylidene)naphthalen-2(1*H*)-one 1**

**^1^H NMR (DMSO-*d6*) spectrum of 3-hydroxy-1-(2-phenylhydrazineylidene)naphthalen-2(1*H*)-one 1**

**^1^H NMR (DMSO-*d6*) spectrum of 3-hydroxy-1-(2-phenylhydrazineylidene)naphthalen-2(1*H*)-one 1**

**^13^C APT NMR (DMSO-*d6*)spectrum of** **3-hydroxy-1-(2-phenylhydrazineylidene)-naphthalen-2(1*H*)-one 1**


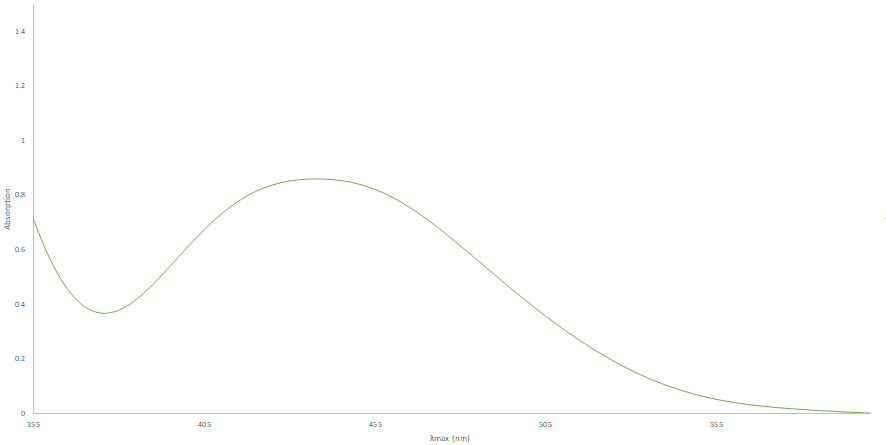


**UV spectrum of** **3-hydroxy-1-(2-(2-nitrophenyl)hydrazineylidene)naphthalene-2(1*H*)-one** **2**


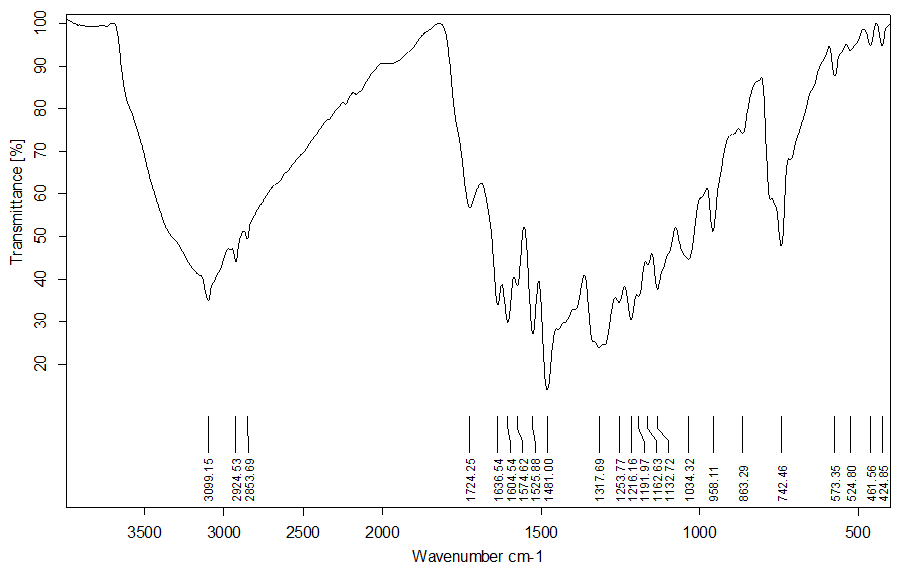


**IR (KBr)spectrum of 3-hydroxy-1-(2-(2-nitrophenyl)hydrazineylidene)naphthalene-2(1*H*)-one** **2**


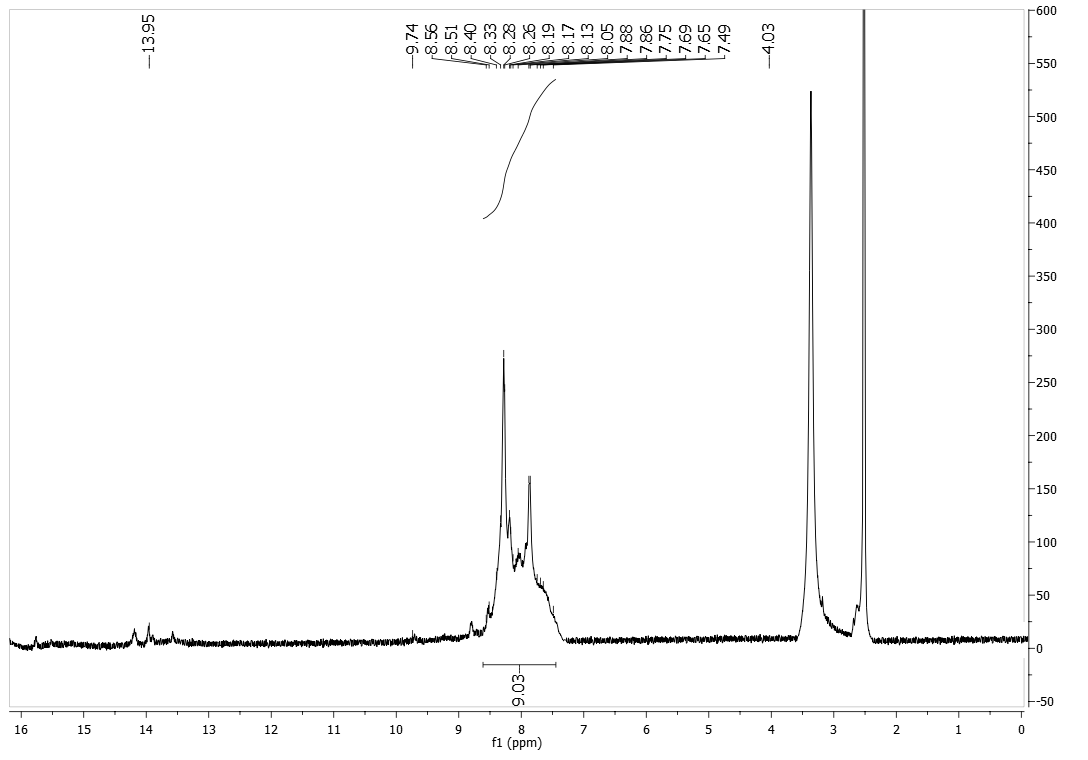


**^1^H NMR (DMSO-*d6*) spectrum of 3-hydroxy-1-(2-(2-nitrophenyl)hydrazineylidene)-naphthalene-2(1*H*)-one** **2**


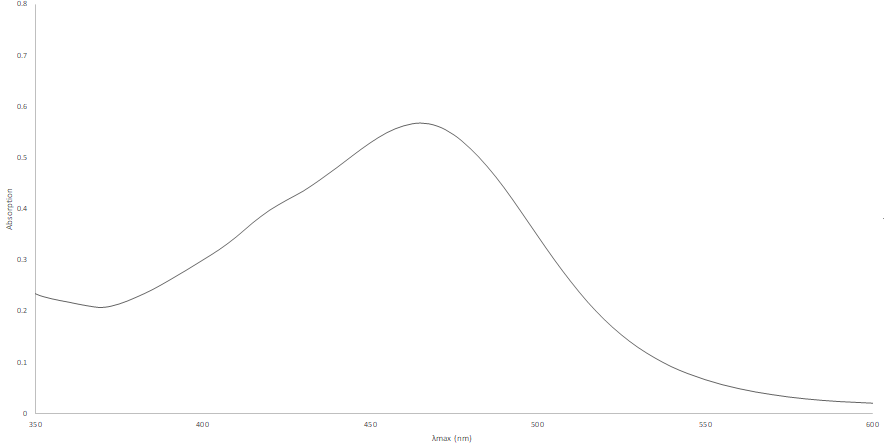


**UV spectrum of** **3-hydroxy-1-(2-(3-nitrophenyl)hydrazineylidene)naphthalen-2(1*H*)-one** **3**


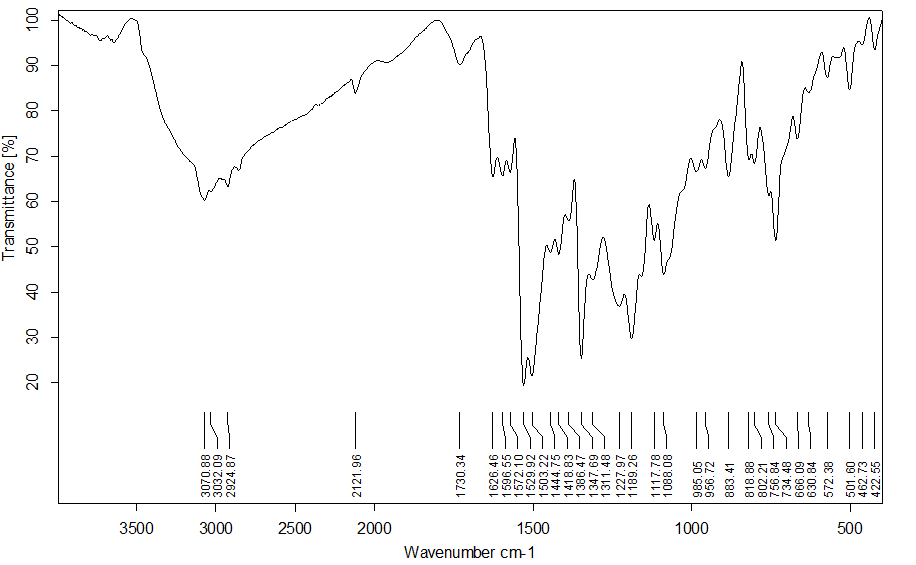


**IR (KBr)spectrum of** **3-hydroxy-1-(2-(3-nitrophenyl)hydrazineylidene)naphthalen-2(1*H*)-one** **3**

**^1^H NMR (DMSO-*d6*) spectrum of 3-hydroxy-1-(2-(3-nitrophenyl)hydrazineylidene)-naphthalen-2(1*H*)-one** **3**

**^13^C APT NMR (DMSO-*d6*)spectrum of 3-hydroxy-1-(2-(3-nitrophenyl)hydrazine-ylidene)naphthalen-2(1*H*)-one** **3**


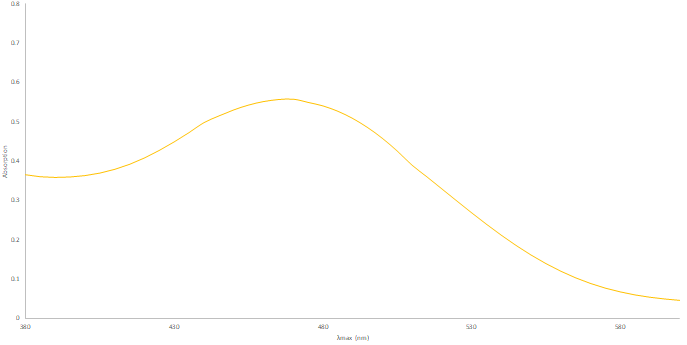


**UV spectrum of 3-hydroxy-1-(2-(4-nitrophenyl)hydrazineylidene)naphthalen-2(1H)-one** **4**


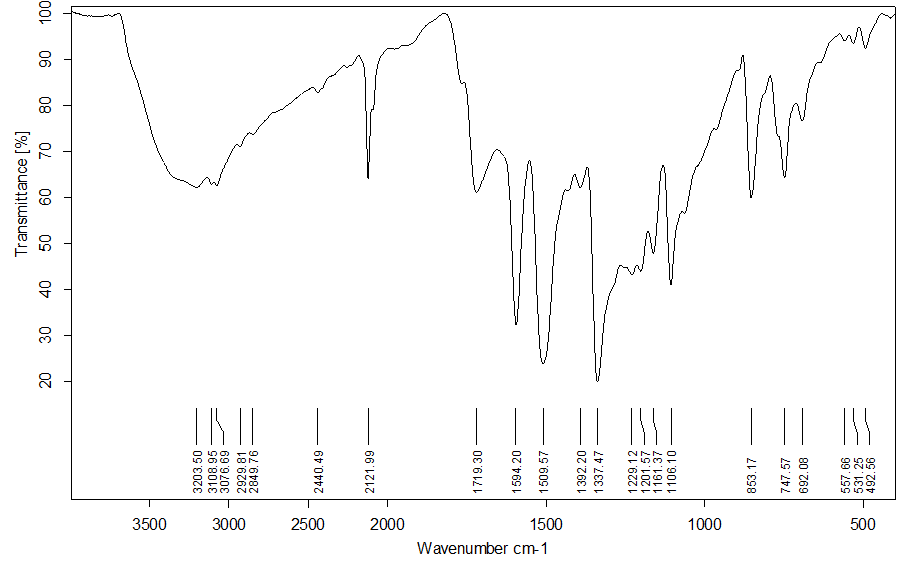


**IR (KBr)spectrum of 3-hydroxy-1-(2-(4-nitrophenyl)hydrazineylidene)naphthalen-2(1H)-one** **4**

**^1^H NMR (DMSO-*d6*) spectrum of 3-hydroxy-1-(2-(4-nitrophenyl)hydrazineylidene)-naphthalen-2(1H)-one** **4**


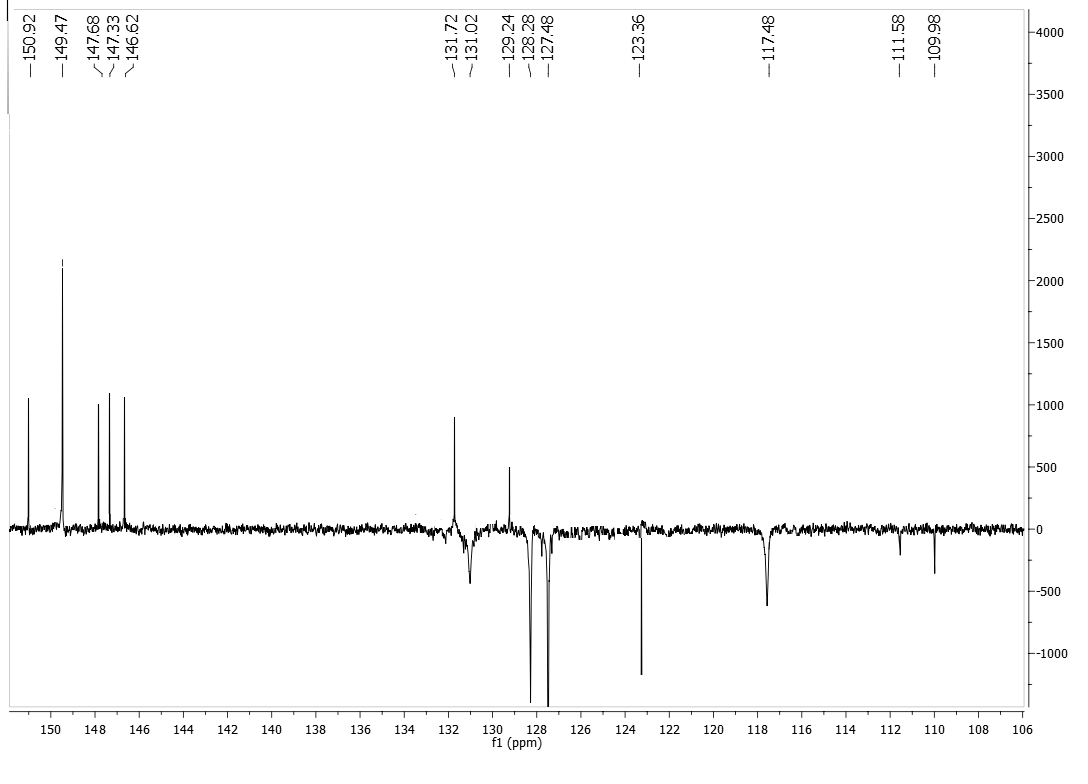


**^13^C APT NMR (DMSO-*d6*) spectrum of 3-hydroxy-1-(2-(4-nitrophenyl)hydrazine-ylidene)naphthalen-2(1H)-one** **4**


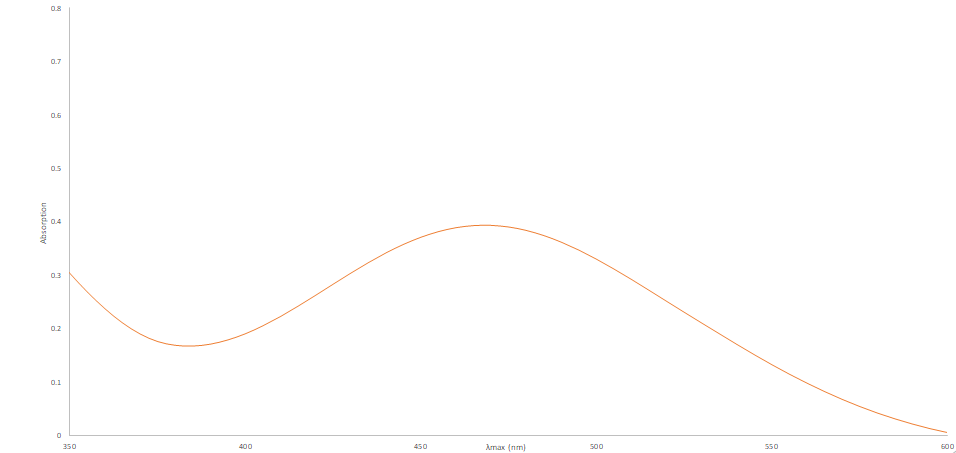


**UV spectrum of 1-(2-(2-chlorophenyl)hydrazineylidene)-3-hydroxynaphthalen-2(1*H*)-one 5**


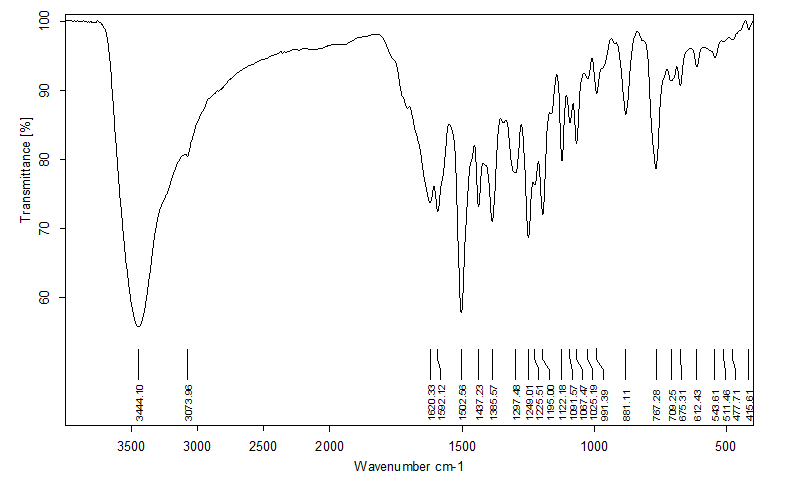


**IR(KBr) spectrum of 1-(2-(2-chlorophenyl)hydrazineylidene)-3-hydroxynaphthalen-2(1*H*)-one 5**

**^1^H NMR (DMSO-*d6*) spectrum of 1-(2-(2-chlorophenyl)hydrazineylidene)-3-hydroxynaphthalen-2(1*H*)-one 5**

**^1^H NMR (DMSO-*d6*) spectrum of 1-(2-(2-chlorophenyl)hydrazineylidene)-3-hydroxynaphthalen-2(1*H*)-one 5**

**^13^C APT NMR (DMSO-*d6*) spectrum of 1-(2-(2-chlorophenyl)hydrazineylidene)-3-hydroxynaphthalen-2(1*H*)-one 5**


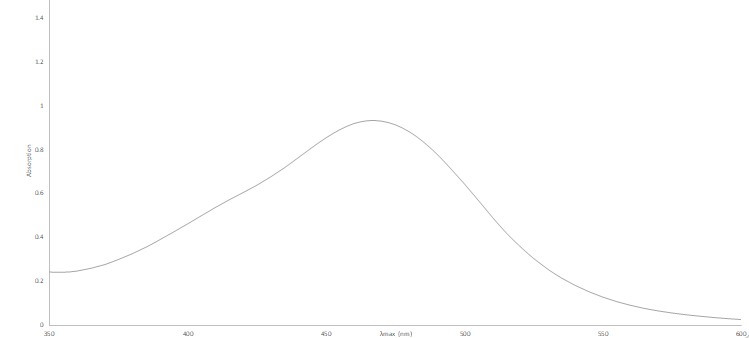


**UV spectrum of 1-(2-(3-chlorophenyl)hydrazineylidene)-3-hydroxynaphthalen-2(1*H*)-one 6**


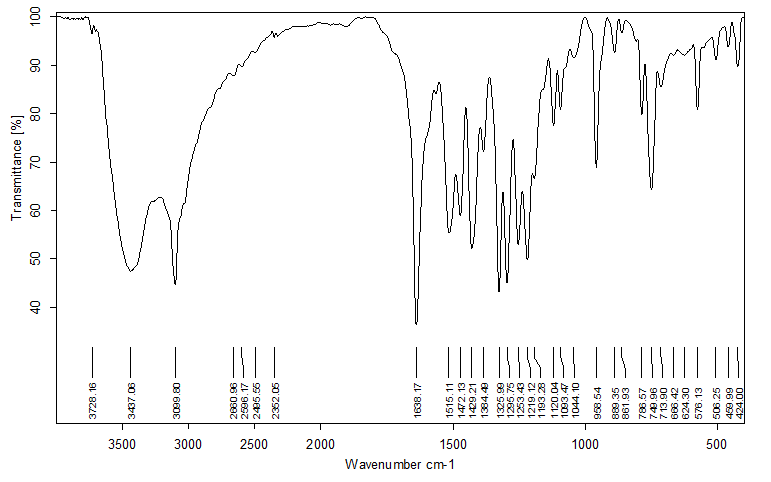


**IR(KBr) spectrum of**  **1-(2-(3-chlorophenyl)hydrazineylidene)-3-hydroxy-naphthalen-2(1*H*)-one 6**

**^1^H NMR (CDCl_3_) spectrum of** **1-(2-(3-chlorophenyl)hydrazineylidene)-3-hydroxynaphthalen-2(1*H*)-one 6**

**^1^H NMR (CDCl_3_) spectrum of** **1-(2-(3-chlorophenyl)hydrazineylidene)-3-hydroxynaphthalen-2(1*H*)-one 6**

**^13^C APT** **NMR (CDCl_3_) spectrum of** **1-(2-(3-chlorophenyl)hydrazineylidene)-3-hydroxynaphthalen-2(1*H*)-one 6**


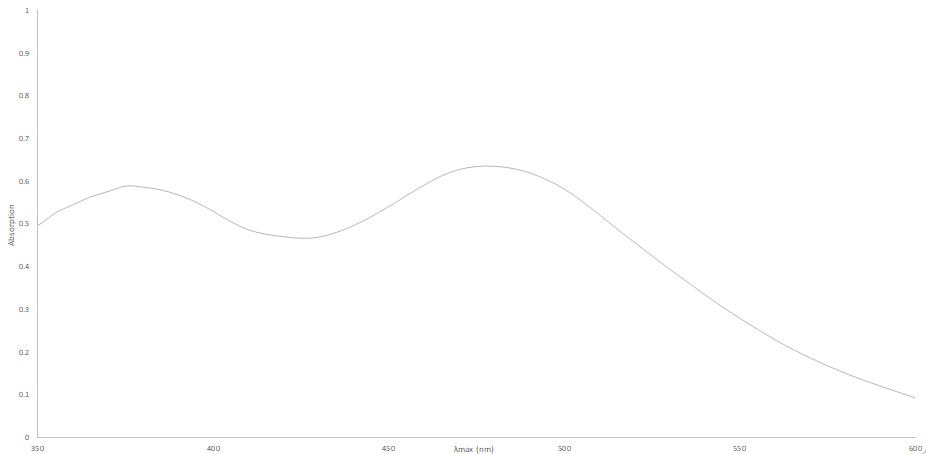


**UV spectrum of 1-(2-(4-chlorophenyl)hydrazineylidene)-3-hydroxynaphthalen-2(1*H*)-one 7**


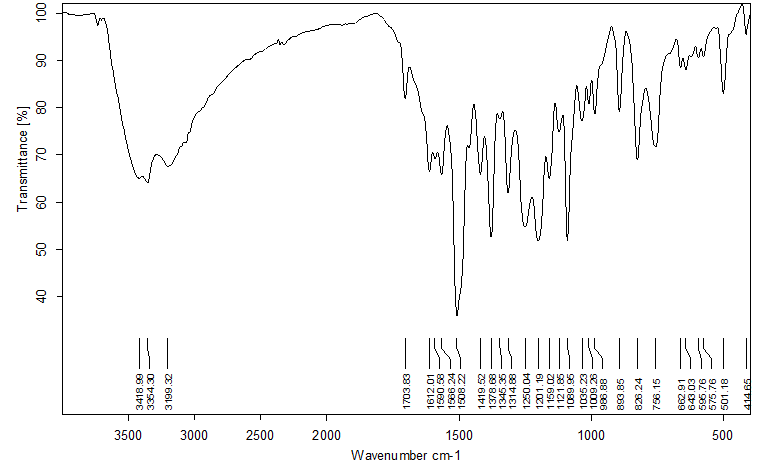


**IR(KBr) spectrum of 1-(2-(4-chlorophenyl)hydrazineylidene)-3-hydroxy-naphthalen-2(1*H*)-one 7**


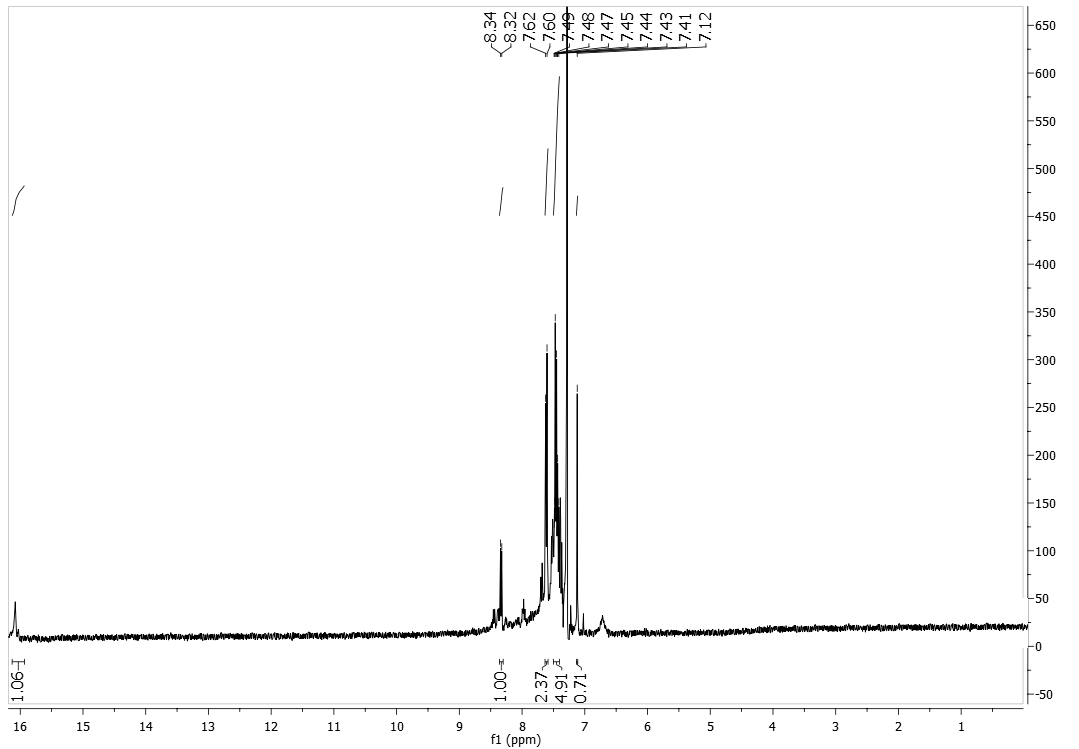


**^1^H NMR (CDCl_3_) spectrum of 1-(2-(4-chlorophenyl)hydrazineylidene)-3-hydroxy-naphthalen-2(1*H*)-one 7**

**^13^C APT** **NMR (DMSO-*d6*) spectrum of 1-(2-(4-chlorophenyl)hydrazineylidene)-3-hydroxy-naphthalen-2(1*H*)-one 7**

**^13^C APT** **NMR (DMSO-*d6*) spectrum of 1-(2-(4-chlorophenyl)hydrazineylidene)-3-hydroxy-naphthalen-2(1*H*)-one 7**


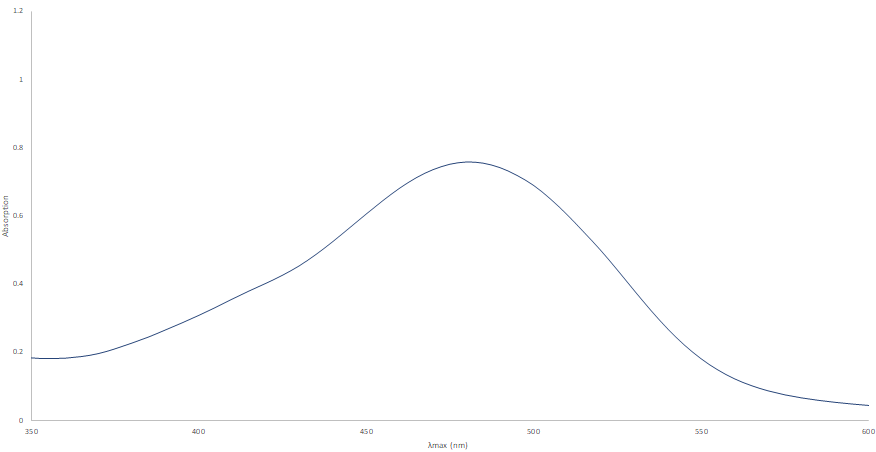


**UV spectrum of 3-hydroxy-1-(2-(2-tolyl)hydrazineylidene)naphthalen-2(1*H*)-one 8**


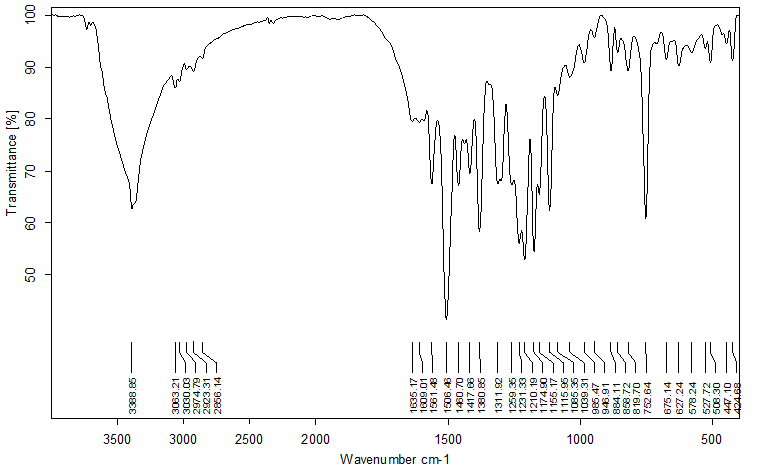


**IR (KBr) spectrum of 3-hydroxy-1-(2-(2-tolyl)hydrazineylidene)naphthalen-2(1*H*)-one 8**

**^1^H NMR (CDCl_3_) spectrum of 3-hydroxy-1-(2-(2-tolyl)hydrazineylidene)naphthalen-2(1*H*)-one 8**


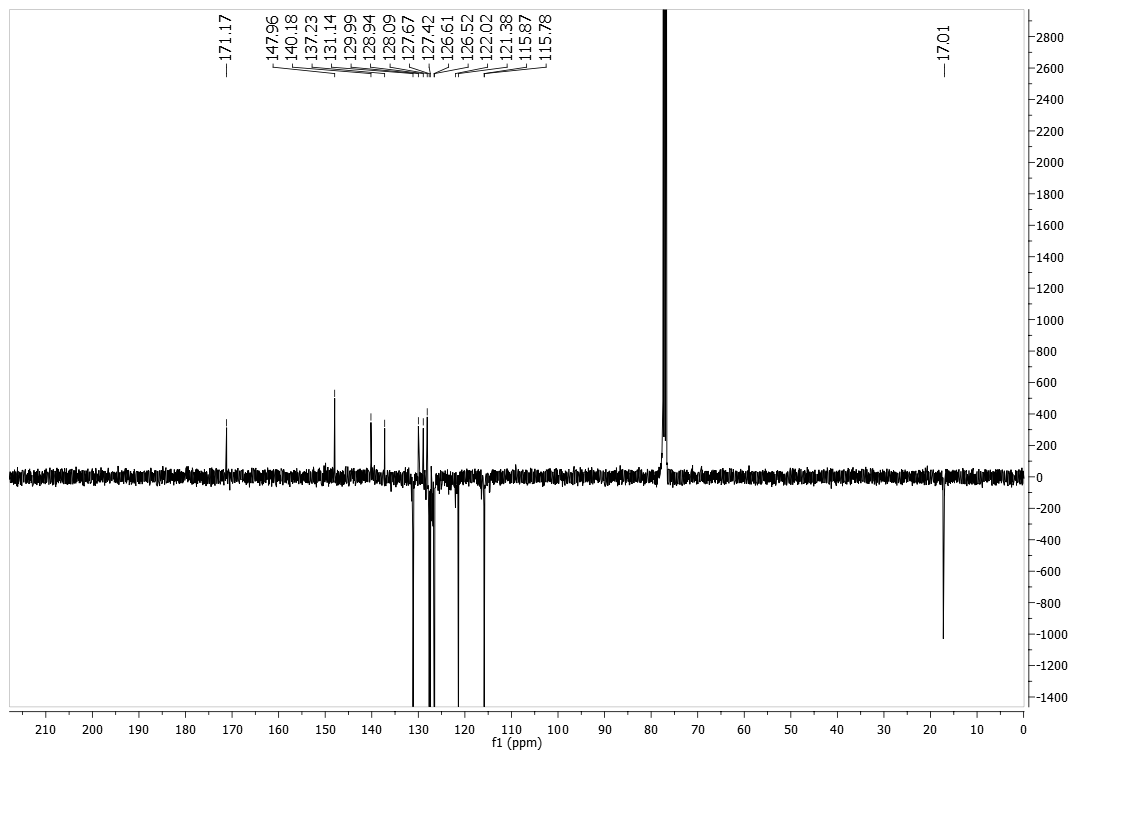


**^13^C APT NMR (CDCl_3_) spectrum of 3-hydroxy-1-(2-(2-tolyl)hydrazineylidene)-naphthalen-2(1*H*)-one 8**


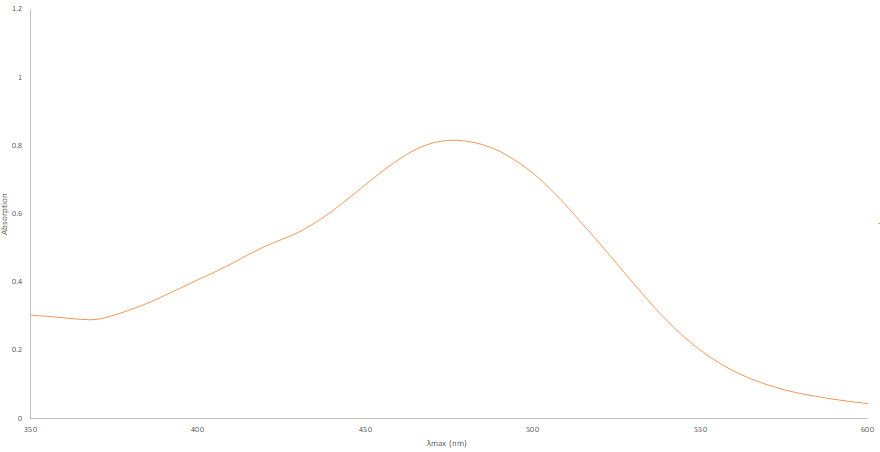


**UV spectrum of 3-hydroxy-1-(2-(3-tolyl)hydrazineylidene)naphthalen-2(1*H*)-one 9**


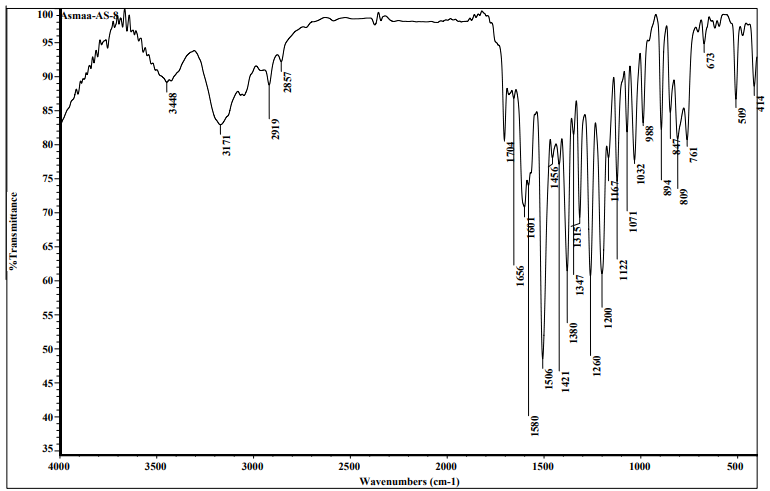


**IR (KBr) spectrum of 3-hydroxy-1-(2-(3-tolyl)hydrazineylidene)naphthalen-2(1*H*)-one 9**

**^1^H NMR (CDCl_3_) spectrum of** **3-hydroxy-1-(2-(3-tolyl)hydrazineylidene)-naphthalen-2(1*H*)-one 9**

**^13^C APT NMR (CDCl_3_) spectrum of** **3-hydroxy-1-(2-(3-tolyl)hydrazineylidene)-naphthalen-2(1*H*)-one 9**


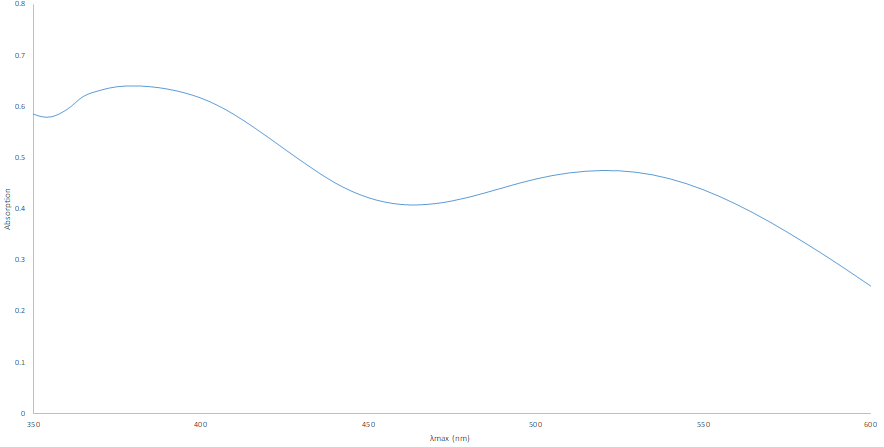


**UV spectrum 3-hydroxy-1-(2-(4-tolyl)hydrazineylidene)naphthalen-2(1*H*)-one 10**


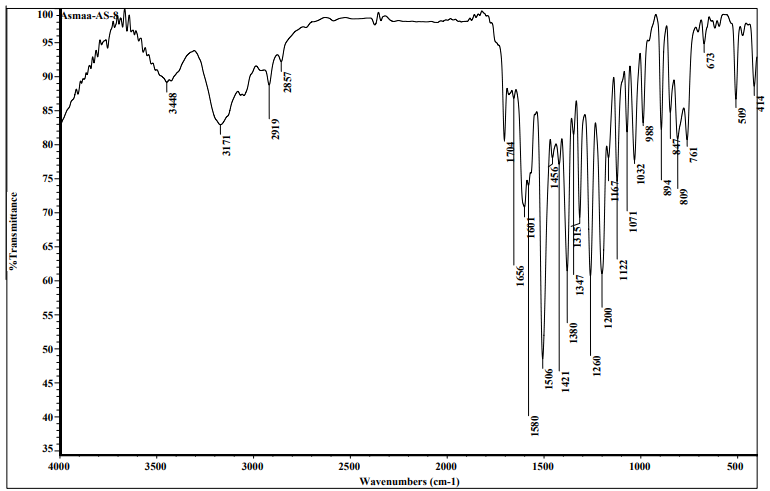


**IR (KBr) spectrum of 3-hydroxy-1-(2-(4-tolyl)hydrazineylidene)naphthalen-2(1*H*)-one 10**


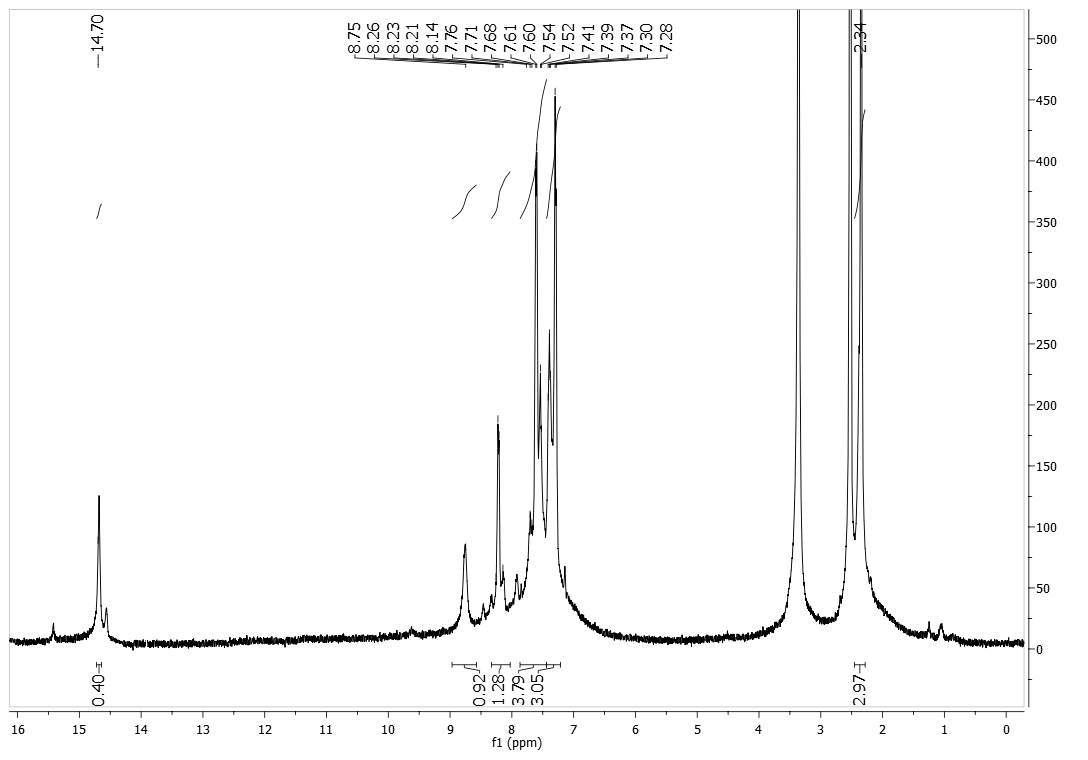


**^1^H NMR (DMSO-*d6*) spectrum of 3-hydroxy-1-(2-(4-tolyl)hydrazineylidene)naphthalen-2(1*H*)-one 10**


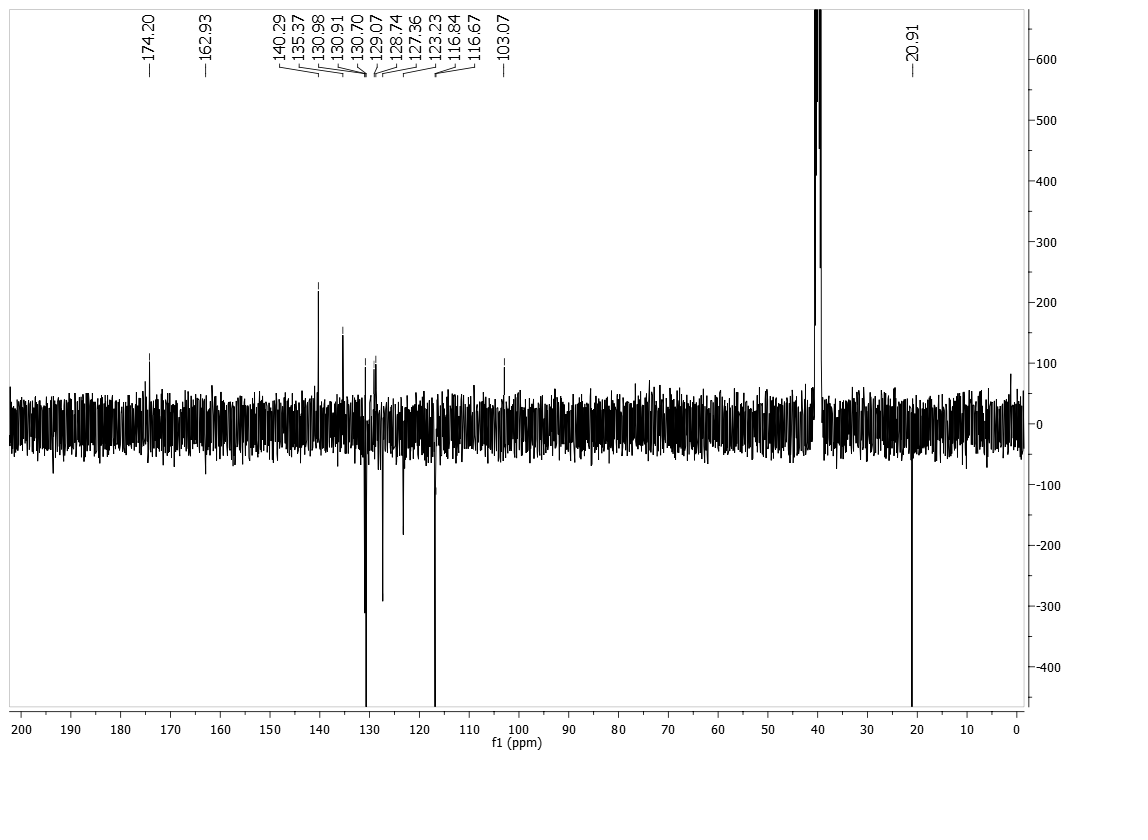


**^13^C APT NMR (DMSO-*d6*) 3-hydroxy-1-(2-(4-tolyl)hydrazineylidene)n-aphthalen-2(1*H*)-one 10**


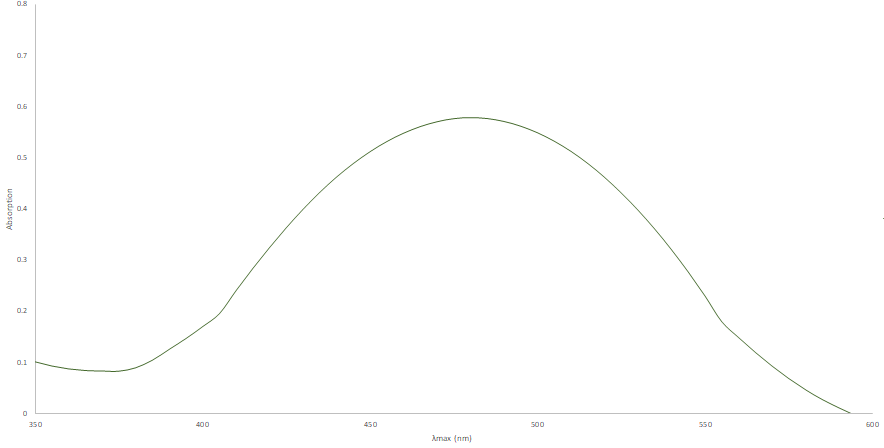


**UV spectrum of 3-hydroxy-1-(2-(2-methoxyphenyl)hydrazineylidene)naphthalen-2(1*H*)-one 11**


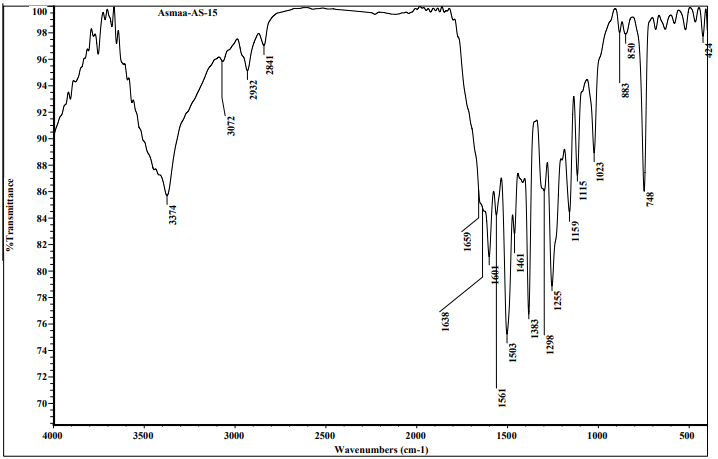


**IR (KBr) spectrum of 3-hydroxy-1-(2-(2-methoxyphenyl)hydrazineylidene)naphthalen-2(1*H*)-one 11**

**^1^H NMR (DMSO-*d6*) spectrum of 3-hydroxy-1-(2-(2-methoxyphenyl)hydrazine-ylidene)naphthalen-2(1*H*)-one 11**


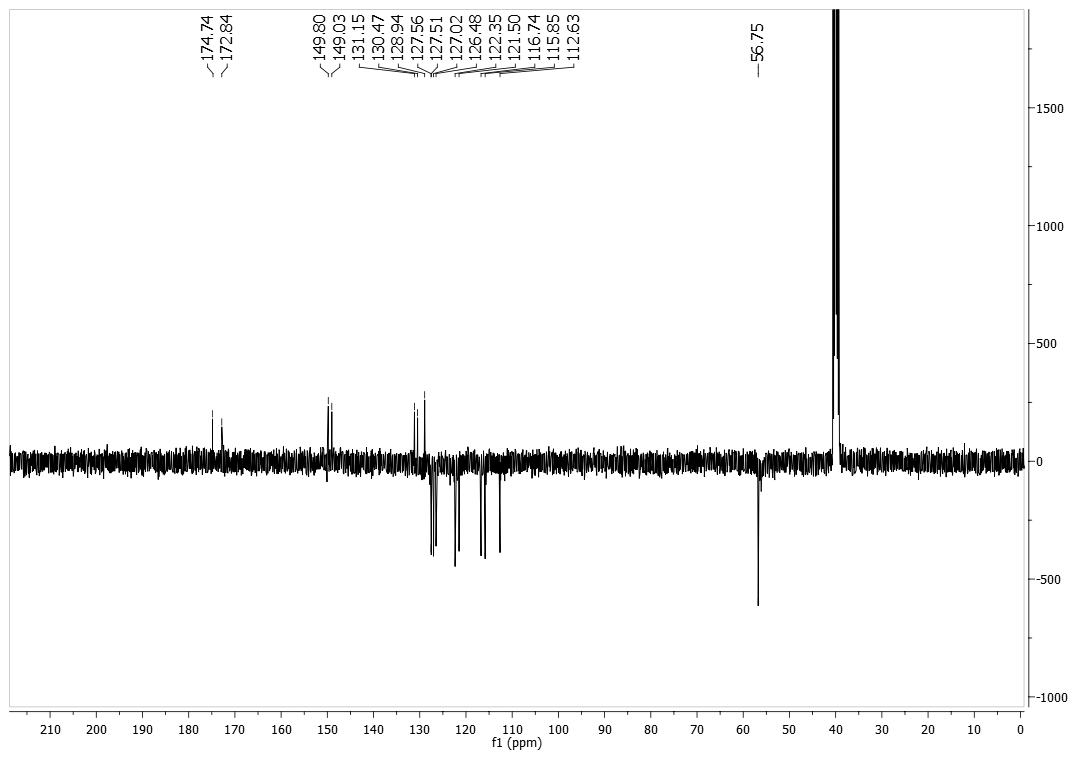


**^13^C APT NMR (DMSO-*d6*) spectrum of 3-hydroxy-1-(2-(2-methoxyphenyl)hydrazine-ylidene)naphthalen-2(1*H*)-one 11**


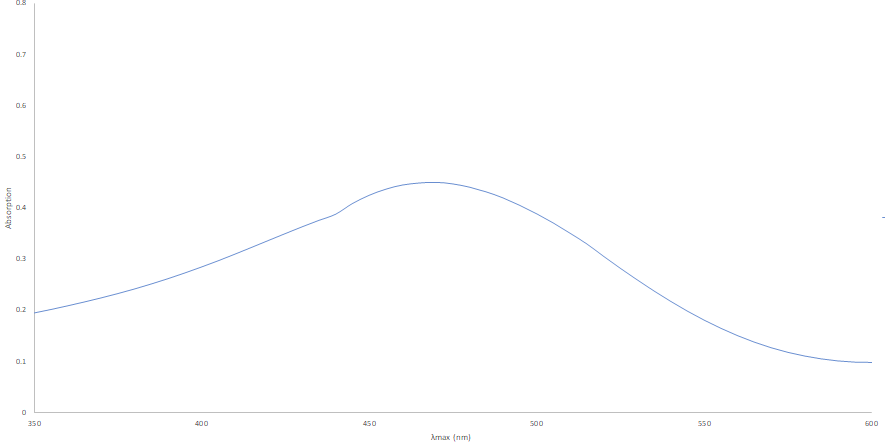


**UV spectrum of 3-hydroxy-1-(2-(4-methoxyphenyl)hydrazineylidene)naphthalen-2(1*H*)-one 12**


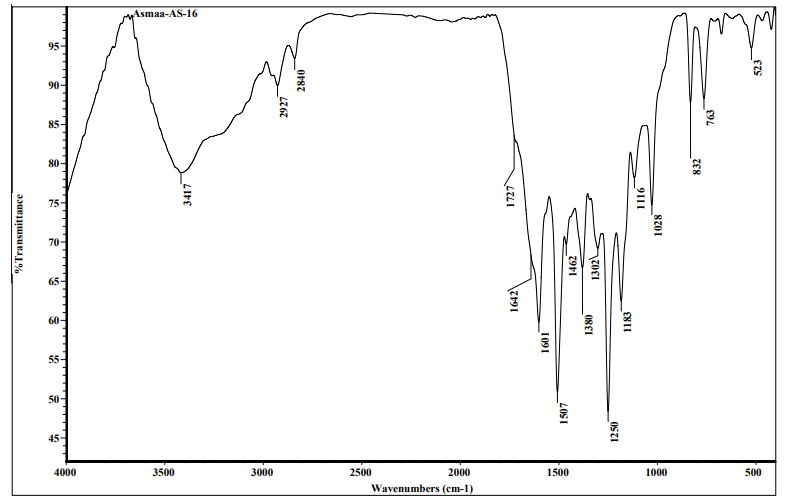


**IR (KBr) spectrum of 3-hydroxy-1-(2-(4-methoxyphenyl)hydrazineylidene)naphthalen-2(1*H*)-one 12**

**^1^H NMR (DMSO-*d6*) spectrum of** **3-hydroxy-1-(2-(4-methoxyphenyl)hydrazine-ylidene)naphthalen-2(1*H*)-one 12**


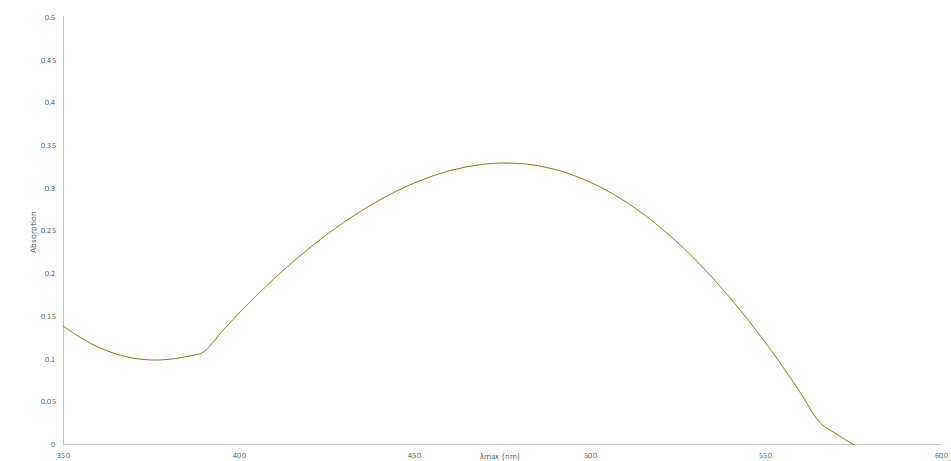


**UV spectrum of 3-hydroxy-1-(2-(2-hydroxyphenyl)hydrazineylidene)naphthalen-2(1*H*)-one 13**


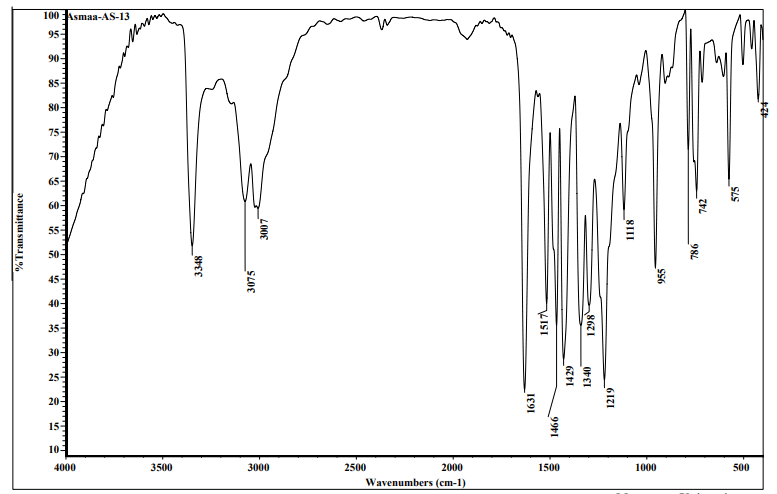


**IR (KBr) spectrum of 3-hydroxy-1-(2-(2-hydroxyphenyl)hydrazineylidene)-naphthalen-2(1*H*)-one 13**

**^1^H NMR (DMSO-*d6*) spectrum of 3-hydroxy-1-(2-(2-hydroxyphenyl)hydrazine-ylidene)naphthalen-2(1*H*)-one 13**

******

**^1^H NMR (DMSO-*d6*) spectrum of 3-hydroxy-1-(2-(2-hydroxyphenyl)hydrazine-ylidene)naphthalen-2(1*H*)-one 13**

**^13^C APT NMR (DMSO-*d6*) spectrum of 3-hydroxy-1-(2-(2-hydroxyphenyl)hydrazine-ylidene)naphthalen-2(1*H*)-one 13**


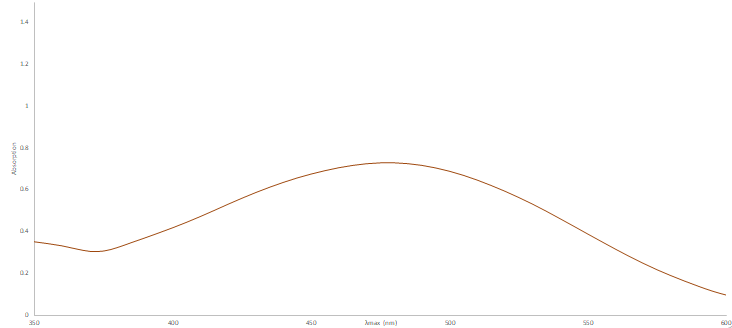


**UV spectrum of 3-hydroxy-1-(2-(4-hydroxyphenyl)hydrazineylidene)naphthalen-2(1*H*)-one 14**


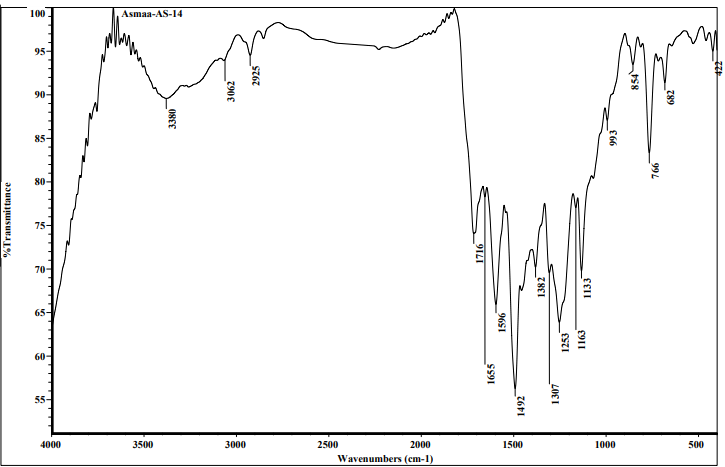


**IR (KBr) spectrum of 3-hydroxy-1-(2-(4-hydroxyphenyl)hydrazineylidene)naphthalen-2(1*H*)-one 14**

**^1^H NMR (DMSO-*d6*) spectrum of 3-hydroxy-1-(2-(4-hydroxyphenyl)hydrazine-ylidene)naphthalen-2(1*H*)-one 14**

**^1^H NMR (DMSO-*d6*) spectrum of 3-hydroxy-1-(2-(4-hydroxyphenyl)hydrazine-ylidene)naphthalen-2(1*H*)-one 14**


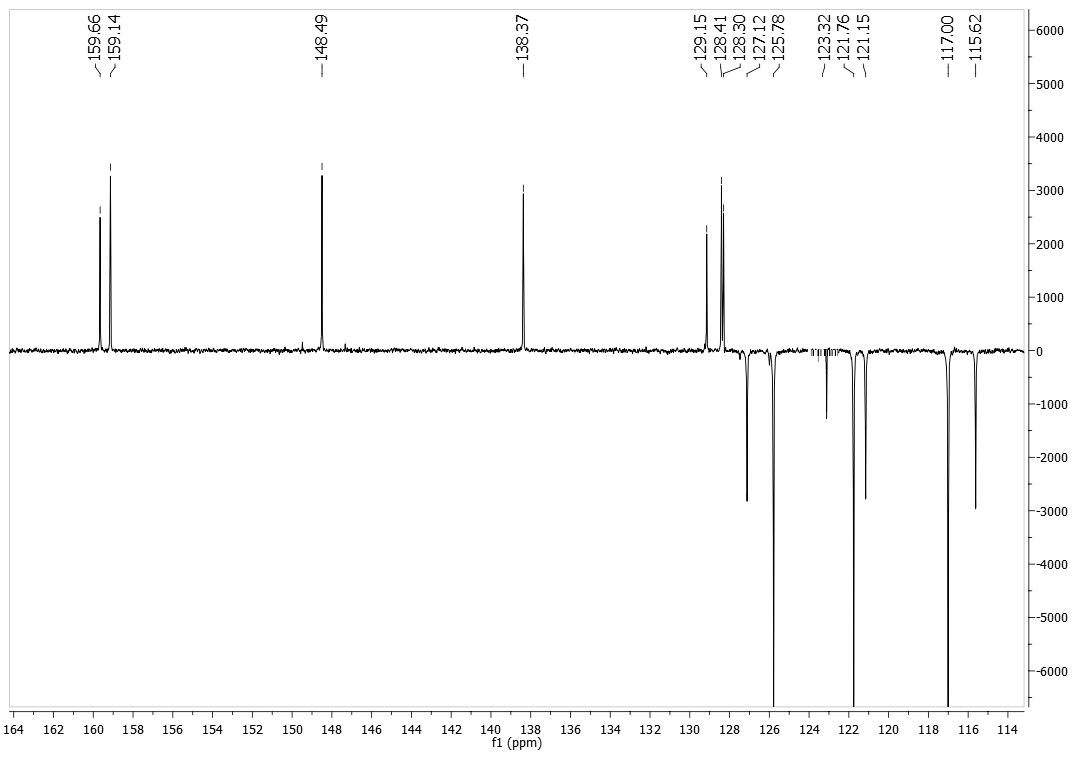


**^13^C APT NMR (DMSO-*d6*) spectrum of 3-hydroxy-1-(2-(4-hydroxyphenyl)hydrazine-ylidene)naphthalen-2(1*H*)-one 14**
